# The Tomato Yellow Leaf Curl Virus Resistance Genes *Ty-1* and *Ty-3* Are Allelic and Code for DFDGD-Class RNA–Dependent RNA Polymerases

**DOI:** 10.1371/journal.pgen.1003399

**Published:** 2013-03-28

**Authors:** Maarten G. Verlaan, Samuel F. Hutton, Ragy M. Ibrahem, Richard Kormelink, Richard G. F. Visser, John W. Scott, Jeremy D. Edwards, Yuling Bai

**Affiliations:** 1 Wageningen UR Plant Breeding, Wageningen University and Research Centre, Wageningen, The Netherlands; 2 Centre for BioSystems Genomics, Wageningen, The Netherlands; 3 Graduate School Experimental Plant Sciences, Wageningen University and Research Centre, Wageningen, The Netherlands; 4 Gulf Coast Research and Education Center, University of Florida, Wimauma, Florida, United States of America; 5 Laboratory of Virology, Wageningen University and Research Centre, Wageningen, The Netherlands; Virginia Tech, United States of America

## Abstract

Tomato Yellow Leaf Curl Virus Disease incited by *Tomato yellow leaf curl virus* (TYLCV) causes huge losses in tomato production worldwide and is caused by different related begomovirus species. Breeding for TYLCV resistance has been based on the introgression of multiple resistance genes originating from several wild tomato species. In this study we have fine-mapped the widely used *Solanum chilense*–derived *Ty-1* and *Ty-3* genes by screening nearly 12,000 plants for recombination events and generating recombinant inbred lines. Multiple molecular markers were developed and used in combination with disease tests to fine-map the genes to a small genomic region (approximately 70 kb). Using a Tobacco Rattle Virus–Virus Induced Gene Silencing approach, the resistance gene was identified. It is shown that *Ty-1* and *Ty-3* are allelic and that they code for a RNA–dependent RNA polymerase (RDR) belonging to the RDRγ type, which has an atypical DFDGD motif in the catalytic domain. In contrast to the RDRα type, characterized by a catalytic DLDGD motif, no clear function has yet been described for the RDRγ type, and thus the *Ty-1/Ty-3* gene unveils a completely new class of resistance gene. Although speculative, the resistance mechanism of *Ty-1*/*Ty-3* and its specificity towards TYLCV are discussed in light of the function of the related RDRα class in the amplification of the RNAi response in plants and transcriptional silencing of geminiviruses in plants.

## Author Summary

Tomato yellow leaf curl virus and related begomoviruses cause major economic damage to tomato production in tropical and subtropical regions around the world. Because cultivated tomato is inherently susceptible to these viruses, breeders have incorporated several resistance alleles from wild tomato relatives. Among these are the commercially important alleles, *Ty-1* and *Ty-3*, which were introgressed from the wild tomato relative *Solanum chilense*. These genes were originally mapped to different regions on chromosome 6, but recent findings suggest they may rather be alleles of the same gene. Here, we describe the precise mapping of *Ty-1* and *Ty-3* to a common chromosomal region, and we show that *Ty-1* and *Ty-3* are alleles that code for an RNA–dependent RNA polymerase of a class for which no function had been described before. Thus, *Ty-1*/*Ty-3* unveils a completely new class of resistance genes. These results will be useful to breeders who utilize these genes in their breeding programs, and further studies should shed new light on the mechanism by which this gene functions.

## Introduction

Plant pathogens are a major limiting factor for agricultural productivity worldwide. Viruses are among these and cause large yield losses in a variety of economically important crops. Although most viruses have small genomes and code for a very limited amount of proteins, they can cause a variety of disease symptoms, and the mechanisms underlying these are still mostly unknown. Plants utilize several lines of defense mechanisms to protect themselves from pathogen invasion. The mechanism that has been studied the most is resistance (R) gene-mediated resistance, which relies on the ability of a plant to recognize a pathogen and consequently trigger the hypersensitive cell death response (HR) [1]. Meanwhile, a large number of R genes have been identified, including ones responsible for the (in)direct recognition of viruses, such as *Sw-5* for tospoviruses in tomato [2], *Rx2* for *Potato virus X* [3] and the *I* locus for *Bean common mosaic virus* [4]. In addition to these dominant R genes, a second type of resistance gene is inherited recessively, which is more common in resistances to viruses compared with resistance to fungi or bacteria [5]–[6]. Most of these genes are linked to the eukaryotic translation initiation complex and negatively affect the viral RNA replication cycle [7].

RNA silencing (also called RNA interference, RNAi), is a conserved eukaryotic gene regulation mechanism that involves the biogenesis of small (s)RNA molecules of ∼21–26 nucleotides in size from perfect or imperfect long double stranded (ds)RNA molecules by an enzyme designated Dicer (mammals, insects), or Dicer-like protein (DCL) (plants) [8]. One strand of these sRNA molecules is incorporated into an RNA-induced silencing complex (RISC) and enables the latter to sense and target RNA molecules with sequence complementarity to the uploaded RNA strand for degradation or translational arrest by means of the core Argonaute (AGO) protein. In recent years, RNA silencing has become known as an antiviral defense mechanism in plants and insects in which viral double-stranded RNA replicative intermediates or secondary RNA folding structures are cleaved into primary, small-interfering (si)RNA molecules. In plants, the viral primary siRNA molecules also act as primers for the host RNA-dependent RNA polymerases (RDR) to convert (aberrant) RNA target sequences into new long dsRNAs. These in turn become processed into secondary siRNAs. This not only leads to an amplification of the siRNA signal, but also results in a distributional spread of siRNA molecules from the entire RNA target sequence, referred to as transitive silencing [9]. The amplification of siRNAs is required to mount a strong antiviral RNAi response. Arabidopsis *RDR1*, *2* and *6*, and orthologs of these genes, have been demonstrated to be involved in this amplification and plants from which these genes have been knocked-out exhibit higher susceptibility to various plant viruses [10]–[14].

The whitefly transmitted tomato yellow leaf curl disease (TYLCD) is one of the most devastating diseases of tomato (*Solanum lycopersicum*) and is caused by several species of the *Begomovirus* genus (*Geminiviridae*) [15]. Tomato yellow leaf curl viruses (TYLCV) are the most widespread and currently rank 3^rd^ among the economically and scientifically most important plant viruses worldwide [16]. They have a single-stranded circular bi-directionally organized DNA genome with six partially-overlapping open reading frames [17]. Because of their limited coding capacity they rely, like most viruses, not only on their own proteins but also on the host cell machinery for their infection cycle [18]. Since the whitefly insect vector is hard to control, breeding TYLCV resistant tomato cultivars provides an attractive strategy to manage TYLCV. All domesticated tomatoes are susceptible to TYLCV, but high levels of resistance were found in several related wild tomato species. Genetic studies have led to the mapping of five TYLCV resistance/tolerance genes which are being exploited for resistance breeding. These genes have different origins: *Ty-2* was introgressed from *S. habrochaites*, *Ty-5* (*ty-5*) was introgressed from *S. peruvianum* while *Ty-1*, *Ty-3* and *Ty-4* all originated from different *S. chilense* accessions [19]–[24]. So far, none of these genes have been cloned and the underlying resistance mechanisms are still unknown. In contrast with classical R-genes none of the resistances to TYLCV described so far are associated with a HR. Moreover, in almost all TYLCV resistant materials, viral replication occurs [25]–[28]. This also holds true for *Ty-1*/*Ty-3*, where in the donors (*S. chilense* LA1969/LA1932) as well as in a commercial line with a *Ty-1* introgression (3761, A.B. Seeds, Ness Ziona, Israel) TYLCV is replicating and detectable [29]–[31], although the level does not exceed more than 10% of that in susceptible tomato cultivars.

Though many loci (i.e. *Ty-1* to *Ty-5*) for TYLCV resistance have been described, the genes conferring resistance have not been identified. Recently, several papers have reported on host genes in a gene network contributing to the resistance originating from *S. habrochaites* [32]–[34]. By differential cDNA library comparisons of susceptible and resistant tomato lines before and after TYLCV inoculation, approximately 70 genes were found to be preferentially expressed in a tomato line with a resistance introgressed from *S. habrochaites*. For three of those, a lipocalin-like protein (*SlVRSLip*), a *Permease I-like protein* and a hexose transporter *LeHT1*, it was shown that their silencing (partly) compromised resistance.

In our previous study we found that *Ty-1* and *Ty-3* map closer than previously reported and that they might be allelic [35]. In the present study *Ty-1* and *Ty-3* are fine mapped, and using a Tobacco Rattle Virus (TRV) induced silencing approach, the genes have been identified and found to be allelic. They code for an RNA-dependent RNA polymerase (RDR) of the γ class, a class of RDRs for which no function is yet described. The role of this new class of resistance genes will be discussed in light of the TYLCV infection cycle.

## Results

### Fine-mapping of *Ty-1* and *Ty-3*

**Figure 1:**
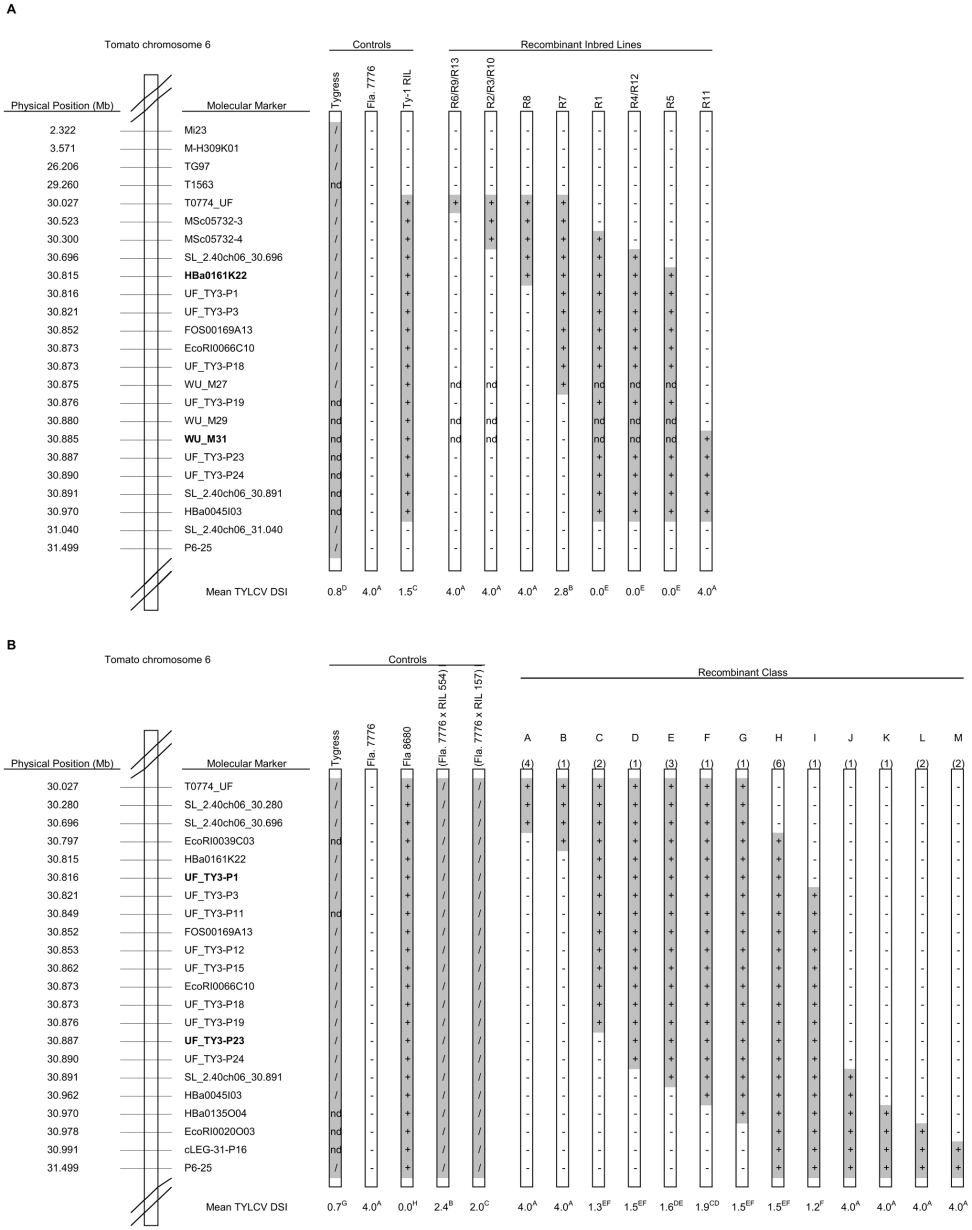
Physical maps showing control lines and introgressed fragments in the RILs used to map *Ty-1* and *Ty-3*. Introgressed segments of the *S. chilense* genome are shaded grey; genotype for each line at each marker is indicated (+ = homozygous *S. chilense*; / = heterozygous; − = homozygous *S. lycopersicum*; nd = not determined). Approximate physical positions are based on the tomato genome assembly SL_2.40, available through the Sol Genomics Network (SGN; http://solgenomics.net/). DSI = mean disease severity index as described in the Materials and Methods; within either population, different superscript letters represent statistically significant differences at P<0.05 based on Duncan's multiple range test. A: Control lines and RILs used for mapping of *Ty-1*. Flanking markers of the *Ty-1* region, HBa0161K22 and WU_M31, are depicted in bold. B: Control lines and RILs used for mapping of *Ty-3*. The number of recombinants recovered in each class is given in parentheses above each recombinant chromosome. Flanking markers, UF_TY3-P1 and UF_TY3-P23 of the *Ty-3* region are depicted in bold.

Previously, we mapped *Ty-1* in the interval between MSc05732-4 and MSc05732-14 [35]. To fine-map *Ty-1*, markers T0774 and SL_2.40ch06_30.891, which flank this interval, were used to screen an F_2_ population derived from a cross between the susceptible Fla. 7776 and a recombinant inbred line (RIL) carrying the *S. chilense Ty-1* introgression. Approximately 2,000 F_2_ plants were screened, 13 recombinants were identified, and RILs were developed for each of these (designated R1 to R13). Four RILs (R1, 4, 12 and 5) containing the *S. chilense* introgression between markers Hba0161K22 and WU_M31 were resistant, while eight RILs that lacked this interval were susceptible (Figure 1A). R7, which resulted from a recombination event between markers WU-M27 and UF_TY3-P19, showed an intermediate response. These results were confirmed for the three most informative recombinants (R7, R8 and R11) (Table S1) using agroinoculation and show that *Ty-1* is located between HBa0161K22 and WU_M31, an interval of approximately 70 kb.

**Figure 2:**
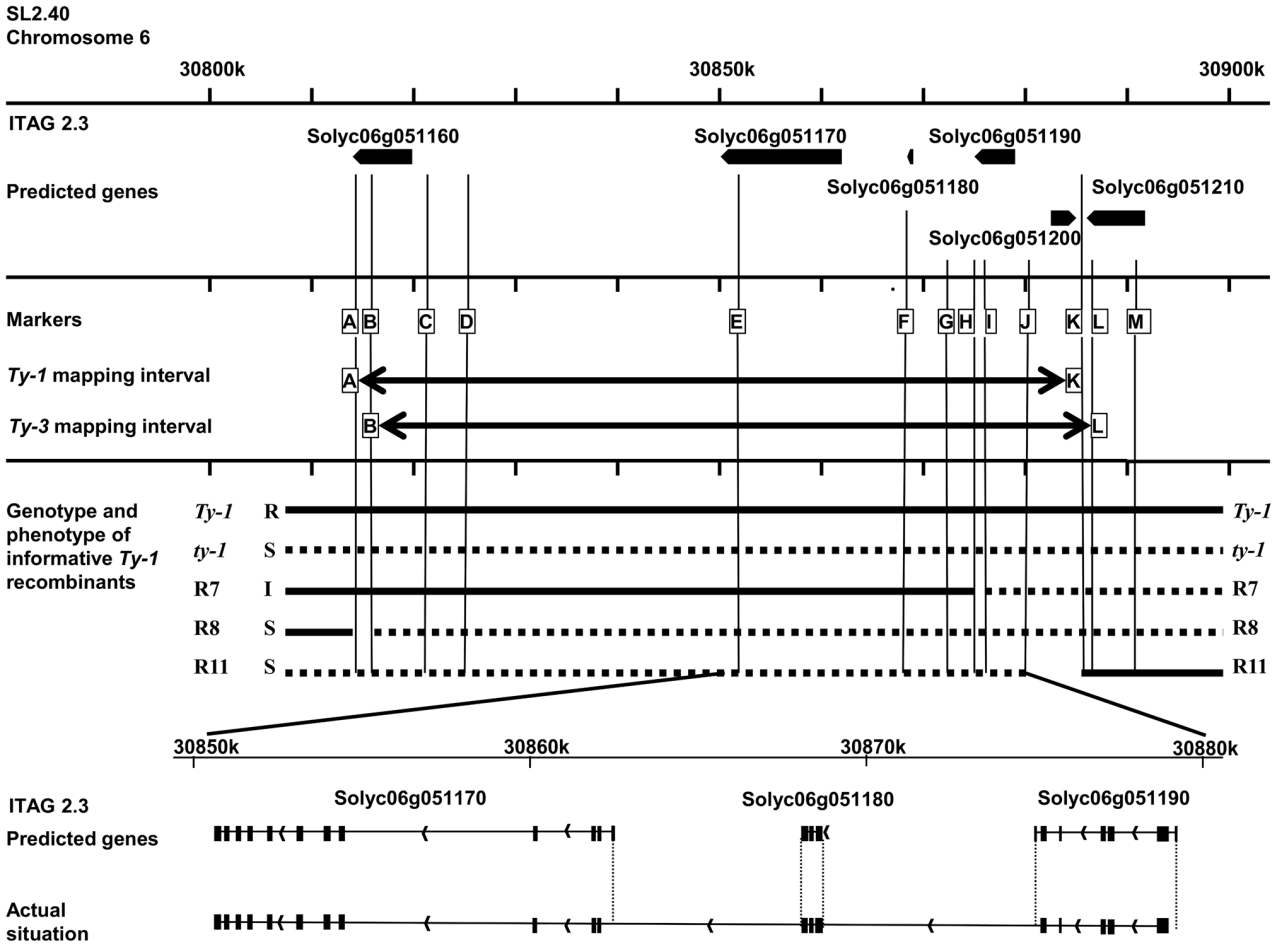
Schematic representation of the region of interest of chromosome 6. Depicted is the region 30,800,000 to 30,900,000 of chromosome 6 with the genomic annotations of the ITAG2.3 release [36]. In the first frame the six predicted genes are represented by arrows. In the next frame the markers used to genotype the recombinants in this study are shown (A = HBa0161K22, B = UF_TY3-P1, C = UF_TY3-P3, D = WU_M17, E = FOS00169A13, F = WUR_M25, G = UF_TY3-P18, H = WU_M27, I = UF_TY3-P19, J = WU_M29, K = WU_M31, L = UF_TY3-P23, M = UF_TY3-P24). In this frame also the *Ty-1* and *Ty-3* intervals with their flanking markers are depicted. The third frame shows the genotype of the informative recombinants used to fine map *Ty-1*, R7, R8 and R11, note that only for R7 the precise recombination point is known (Figure S2). Also their phenotype upon TYLCV challenge inoculation is shown (Resistant (R), Susceptible (S) and Intermediate (I)). The last frame shows the predicted splicing of gene Solyc06g051170, Solyc06g051180 and Solyc06g051190 compared with the actual situation; differences are indicated with dotted lines.

The *Ty-3* gene was previously mapped between T0774 and T1079 [21]. By screening an F_2_ population (n = 717) from a cross between the susceptible line Fla. 7781 with the resistant line Fla. 8680 (carrying the *Ty-3* introgression from *S. chilense* LA2779), 30 recombinants were identified. RILs of these recombinants were generated and tested with TYLCV. Results mapped *Ty-3* to the interval between T0774 and P6-25 (Table S2). To further narrow down the *Ty-3* interval, RILs of two key recombinants were used to generate three F_2_ sub-populations, A, B and C. Screening more than 10,500 individuals of these sub-populations with markers Mi23 and P6-25 (sub-population A and B) and markers T0774 and T0834 (sub-population C) identified 309 recombinants (Table S3). Cuttings of these recombinants were evaluated for TYLCV disease severity (Table S3; control experiments, Table S4) and interval QTL mapping confirmed the location of *Ty-3* between markers T0774 and P6-25, with a LOD of over 50 in an interval between markers SL_2.40ch06_30.696 and cLEG-31-P16 (Figure S1). Recombinants in this interval were further analysed by testing their RILs with TYLCV and by saturating this region with additional molecular markers (Figure 1B, Table S5). RILs carrying the *S. chilense* LA2779 introgression between markers UF_TY3_P1 and UF_TY3_P23 were resistant (recombinant class C to I, Figure 1B), while RILs with introgressions that did not span this region were susceptible; these results map *Ty-3* to a region of approximately 71 kb that overlaps the region containing *Ty-1* (Figure 2).

### Candidate genes for *Ty-1* and *Ty-3*

According to the ITAG2.3 release of the tomato genome, the region to which *Ty-1*/*Ty-3* mapped was predicted to contain five genes; Solyc06g051160 (408 bp), Solyc06g051170 (1728 bp), Solyc06g051180 (438 bp), Solyc06g051190 (957 bp) and Solyc06g051200 (843 bp) [36] (Figure 2). While gene Solyc06g051160 has an unknown function and Solyc06g051200 encodes a predicted ribosomal protein, the other three genes are each predicted to encode (parts of) an RNA-dependent RNA polymerase (RDR). *Arabidopsis thaliana* RDRs in general are approximately 3 kb in size, but these three predicted genes are all much shorter. Since the genes only share low sequence similarity they likely are not paralogous. Interestingly, the crossing-over event in the intermediate resistant R7 occurred within the candidate gene Solyc06g051190. After amplification and sequence analysis of this gene from R7 and subsequent alignment to the corresponding regions of a *Ty-1* line and a *ty-1* line, the recombination site in R7 could be pinpointed between two SNPs. This region covered less than 100 base pairs in which the recombination point mapped to the last part of predicted exon number 4 (Figures S2 and S3). Plants of R7 thus contained a chimeric predicted gene Solyc06g051190.

### Silencing of Solyc06g051180 and Solyc06g051190 compromises resistance

**Figure 3:**
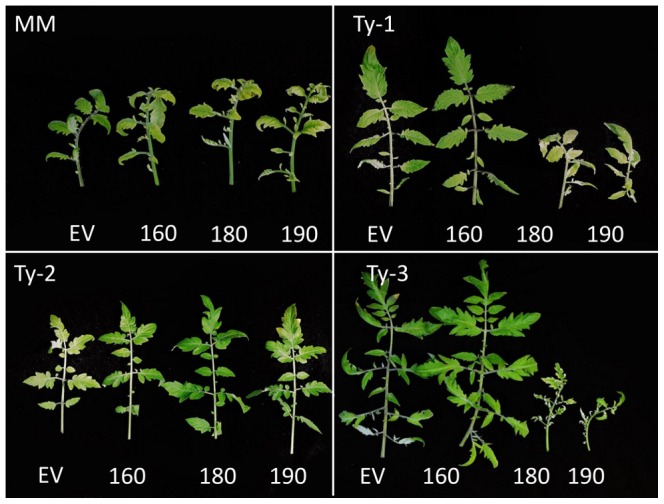
Silencing with constructs TRV2-180 and TRV2-190 compromises TYLCV resistance in *Ty-1* and *Ty-3* lines. Depicted are leaves of plants 6 weeks after inoculation of the TRV silencing constructs and 4 weeks after TYLCV challenge inoculation. EV, empty vector control; 160, TRV2-160; 180, TRV2-180; 190, TRV2-190. All Moneymaker (MM) plants are susceptible, constructs TRV2-180 and TRV2-190 compromise resistance in *Ty-1* and *Ty-3* carrying lines but not in a line with a *Ty-2* introgression.

To identify the *Ty-1* gene from the five candidate genes predicted in the *Ty-1* interval, a Tobacco Rattle Virus (TRV) based Virus Induced Gene Silencing (VIGS) approach was applied. For three out of five genes a VIGS construct could be made; TRV2-160 for Solyc06g051160; TRV2-180 for Solyc06g051180 and TRV2-190 for Solyc06g051190. The two VIGS vectors, TRV2-180 and TR2-190, are specific and both are assumed to target an individual RDR, due to low sequence similarity between Solyc06g051180 and Solyc06g051190. Several attempts to make a VIGS construct for Solyc06g051170 and Solyc06g051200 failed so experiments were done with the available constructs. When plants containing *Ty-1* were agroinfiltrated with empty vector control (EV, TRV2 without an insert) or TRV2-160, and two weeks later superimposed with a TYLCV challenge, the plants maintained resistance to TYLCV. However, when either TRV2-180 or TRV2-190 was used, the resistance was compromised as observed by the appearance of TYLCV disease symptoms (Figure 3). Repeated analysis confirmed these results, which, together with the fact that both Solyc06g051180 and Solyc06g051190 are predicted RDRs located in close proximity to one another within the *Ty-1*/*Ty-3* region, suggest that Solyc06g051180 and Solyc06g051190 might belong to one and the same gene.

### *Ty-1* and *Ty-3* are allelic

Our initial mapping studies indicated that *Ty-1* and *Ty-3* could be alleles of the same gene [35], and the fine mapping of both genes to a similar marker interval strengthened this hypothesis. To test this, the *Ty-1* VIGS approach was again applied to compromise TYLCV resistance in plants carrying the *Ty-3*; as a control, plants with resistance based on *Ty-2* were included. As in the *Ty-1* plants, resistance in the *Ty-3* lines was compromised by TRV2-180 and TRV2-190, but not by TRV-160 (Figure 3). On the other hand, plants containing *Ty-2* remained fully resistant against TYLCV after silencing with all three constructs. Altogether these data indicate that *Ty-1* and *Ty-3* indeed are allelic, while *Ty-2* belongs to another class of resistance genes.

### Solyc06g051170, Solyc06g051180, and Solyc06g051190 together code for *Ty-1* and *Ty-3*

To test the hypothesis that Solyc06g051170, Solyc06g051180 and Solyc06g051190 were part of the same gene, and to clone the entire *Ty-1* gene, several primer pairs were designed to enable RT-PCR amplification of the exons from the three predicted genes, and tested on cDNA of *Ty-1* lines and TYLCV susceptible cv. Moneymaker. Primers designed on the start and stop codons of the three predicted genes did not amplify any products. However, when primers were used that were located a bit downstream of the start codon or upstream of the stop codon products were amplified, indicating that the predicted start and stop codons were wrong. To test whether the initially predicted genes were all part of one RDR-encoding ORF other primer pairs were tested. When primers targeting Solyc06g051170 were combined with Solyc06g051190 (Figure S4, F6-R4) surprisingly a product of approximately 700 bp was amplified indicating that all three predicted genes were indeed not paralogous but part of one and the same RDR gene. This was confirmed by sequence analysis of all overlapping PCR fragments obtained (Figure S4). Using a GeneRacer (Invitrogen) approach the genuine start and stop codons of the RDR gene were identified. Based on these sequences new primers (Table S6, Ty-F7-CACC and Ty-R5) were designed that supported the amplification of a product of approximately 3.1 kb from cDNA of a *Ty-1* line, a *Ty-3* line and from cv. Moneymaker.

### *Ty-1* and *Ty-3* are RDR3/4/5 homologues

Sequence analysis of the amplified *Ty-1*/*Ty-3* gene products revealed that the gene contained 19 exons. Compared with the three predicted genes the first predicted exon of Solyc06g051190 was not expressed, nor was the last exon containing the stop codon (Figure 2). For Solyc06g051180 the first exon started earlier than predicted, the last exon was shorter than predicted, again losing the stop codon. Finally for Solyc06g051170 the first predicted exon was not expressed. Alignment of the amino acid (aa) sequences of *Ty-1*, *Ty-3* and *ty-1* (the susceptible allele from tomato cv. Moneymaker) revealed high sequence identity between all alleles, with only small differences. The most significant difference was a four aa deletion in the N-terminal domain of the susceptible allele. In addition, 20 aa changes were observed, with only small differences between *Ty-1* and *Ty-3*.

**Figure 4:**
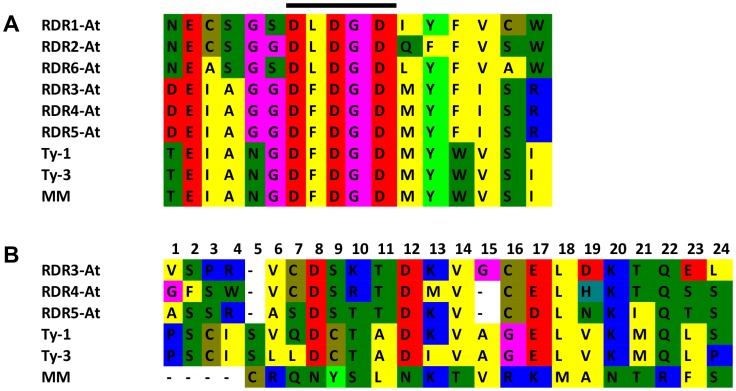
Catalytic domain and polymorphism of *Ty-1* and *Ty-3*. A: Alignment of the catalytic domain and 6 amino acids up- and downstream of the *A. thaliana* RDR1-6, *Ty-1*, *Ty-3* and Moneymaker (MM) alleles. The catalytic domain is indicated with a black bar above the alignment. B: All 24 polymorphisms between Moneymaker and *S. chilense* are shown. The RDR3-5 *A. thaliana* amino acids depicted are taken from a Clustel W alignment (Figure S5 and S6).

**Figure 5:**
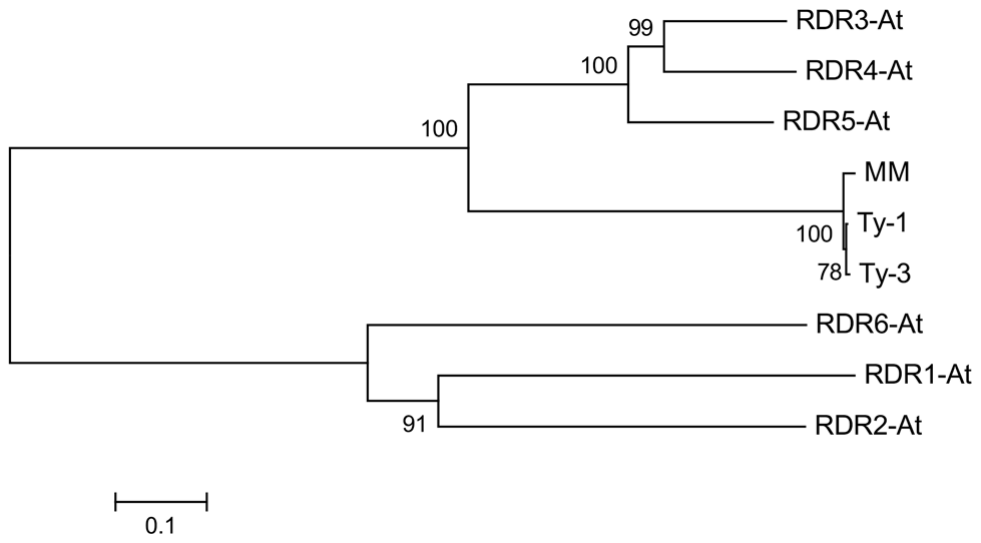
Neighbour joining tree of protein sequences of *A. thaliana* RDR1-6, *Ty-1*, *Ty-3*, and the susceptible Moneymaker allele (MM).

Multiple sequence alignment with the six RDRs identified in *A. thaliana* (Figure S5 and S6) showed a high sequence similarity to RDR3, RDR4, and RDR5 and the presence of the atypical DFDGD catalytic motif of these genes in both *Ty-1* and *Ty-3* alleles (Figure 4A). The homology inferred from the sequence similarity was supported by a phylogenetic analysis using an unrooted neighbor joining tree, in which *Ty-1* and *Ty-3* grouped in the clade containing RDR3, 4 and 5 (Figure 5). Interestingly, although the *ty-1* allele (Moneymaker) appeared in the same clade, it showed less similarity to RDR3/4/5 then the *Ty-1*/*Ty-3* allele (Figure 4B).

### *Ty-1* is relatively high expressed

**Figure 6:**
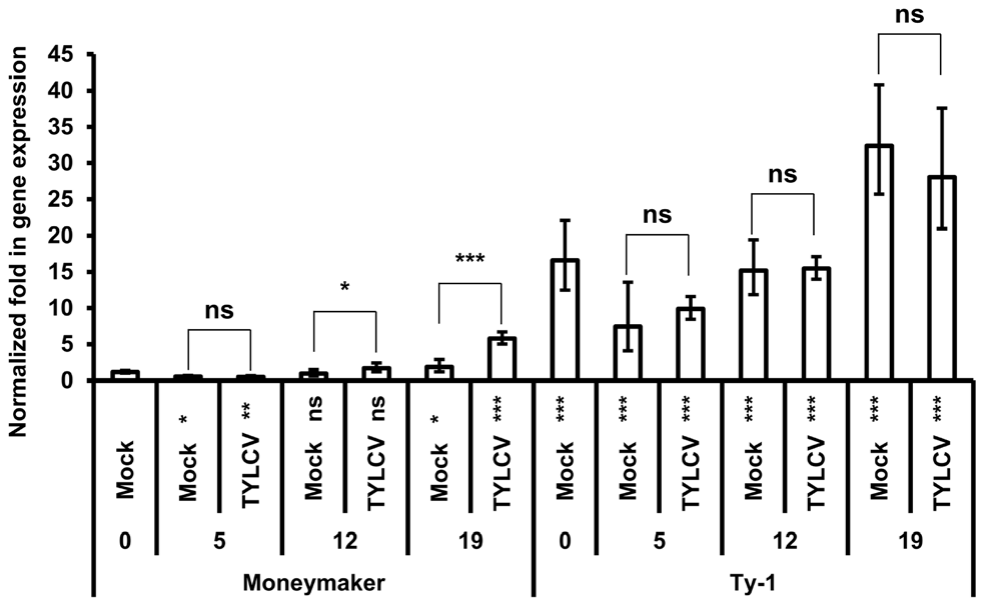
*Ty-1* expression is elevated in resistant lines. Normalized fold in gene expression of the *ty-1* (susceptible) and *Ty-1* (resistant) allele. Numbers below x-axis indicate days after inoculation. Values are normalized against the Moneymaker day 0 sample, bars represent means and standard deviation of five biological replicas. Asterisks under the x-axis represent significant differences to the Moneymaker day 0 sample, asterisks above the bars represent significant differences between Mock or TYLCV treatment. (* = P<0.05, ** = P<0.01, *** = P<0.001, ns = not significant).

Considering a potential role of the *Ty-1* encoded RDR in mounting a strong antiviral RNAi response, its transcriptional expression level was analyzed. To this end, a time-series experiment was performed during which expression of the resistant *Ty-1* and the susceptible *ty-1* allele was quantified upon TYLCV-challenge via agroinoculation in both tomato lines. The expression level of the specific allele was measured by qPCR at several time points (Figure 6). The results showed that at all time points the basic transcription level of the *Ty-1* allele was significantly higher compared to the *ty-1* allele. In the resistance line, no significant difference was observed for the *Ty-1* expression between mock and TYLCV inoculated plants at all time points. However, in the susceptible Moneymaker line, the expression of the *ty-1* allele was induced upon TYLCV inoculation at 12 and 19 days. Compared with day 0 of resistant and susceptible lines, the respective expression of *Ty-1* and *ty-1* was decreased at day 5 and increased at day 19.

## Discussion

Nowadays many dominant and recessive virus resistance genes are well characterized and used in breeding of various crops. Most of these genes either do not allow/prevent viral replication or limit this to the first cells of entry in the host. The TYLCV resistance genes *Ty-1* and *Ty-*3 are different from these because they lead to a level of virus tolerance (rather than immunity). Plants carrying these genes and challenged by the virus still show low levels of viral replication and systemic spread but with moderate (as with *Ty-3*) or no (as with *Ty-1*) visual symptoms. Recently we observed that the *S. chilense* LA1969 derived *Ty-1* and the *S. chilense* LA2779 derived *Ty-3* map close to each other and that they might be allelic [35]. Here we show by fine mapping and functional analysis that *Ty-1* and *Ty-3* are alleles of the same gene and code for RNA-dependent RNA polymerases from a class of functionally unknown RDR genes.

Sequence data shows that most of the SNPs that are present in *Ty-1* are also present in *Ty-3*, which is logical since both alleles originate from *S. chilense* accessions. The most striking difference between *Ty-1*/*Ty-3* and the *ty-1* allele is a deletion of 4 amino acids in the first amino-terminal part of the protein. However, it is not likely that this deletion solely causes a functional loss, since recombinant R7 contains a chimeric RDR – with the N-terminal part of *ty-1*, and still confers partial resistance to TYLCV.

Recently, three genes have been reported which are involved in different networks related to TYLCV resistance introgressed from *S. habrochaites* [32]–[34]. Of the three identified genes, *SlVRSLip* functions downstream *LeHT1* within the same network, while *Permease I-like protein* functions in a different network [32]–[34]. In another study, 18 host genes with a potential role in Tomato Yellow Leaf Curl Sardinia Virus (TYLCSV) infection were identified. Interestingly, almost half of these genes had a role in posttranslational modifications [37]. Whether RDRs encoded by *Ty-1* and *Ty-3* play a role in one any of these networks remains to be analysed.

RDRs are defined by a conserved catalytic domain and are found in RNA viruses and multicellular organisms (plants, fungi and invertebrate animals), but so far are not described in vertebrates and insects. For RNA viruses, the RDR is required to enable replication of its RNA genome to render viral progeny [38]. In multicellular organisms, three major classes of eukaryotic RDRs have been described and some of their functions have been unraveled. The first class is presented by RDRα and members of these are found in plants, animals and fungi. The class of RDRβ genes has been found only in animals and fungi while RDRγ members are only found in plants and fungi [39]. In the model plant *A. thaliana* a total of six RDRs have been identified [40]. Three of them belong to the RDRα type, i.e. *RDR1*, *RDR2* and *RDR6*, and are characterized by a catalytic DLDGD motif. The other three belong to the RDRγ class of genes and are denoted *RDR3*, *RDR4* and *RDR5* (also referred to as *RDR3a*, *RDR3b* and *RDR3c*, respectively). Members of this class have an atypical DFDGD motif in the catalytic domain [40].

The RDRα genes are all known to be involved in RNA silencing, specifically in the amplification of the siRNA signal and resulting in transitive silencing. RNA silencing is generally accepted as a defense system against viral invasion, and is induced by viral dsRNA replicative intermediates or folding structures [41]. Geminiviruses are also targeted by RNAi, as observed by the synthesis of geminivirus-specific siRNAs, (small-RNA directed) viral DNA methylation and post-transcriptional gene silencing of the protein-coding genes [42]–[45]. Although geminiviruses contain a single stranded DNA genome, siRNAs have been observed to originate from the entire virus genome although their distribution was not always equal. The siRNAs are postulated to originate in two ways; 1) as a result of DCL processing from dsRNA molecules that are generated by RDR from bidirectional geminivirus transcripts with overlapping 3′ ends, and 2) mRNA folding structures [42]–[43], [45]–[46].

It is proposed that plants employ silencing of DNA by RNA-directed methylation as a strategy to repress geminivirus replication/transcription [47]. This is supported by two major observations; methylation of geminivirus DNA greatly reduces its ability to replicate in protoplasts [48], and the identification of geminivirus RNA silencing suppressor proteins (RSS) C2, C4 and V2 that exert their activity by interference in the process of DNA methylation and transcriptional gene silencing [49]–[56]. Antiviral RNAi defense against geminiviruses thus seems to mostly rely on a methylation-based defence, a process that involves the action of siRNA-directed methylation pathway component Ago4. Although several studies have pointed towards the involvement of RDR1 and RDR6 in the biogenesis of geminivirus-specific siRNAs, the involvement of other antiviral RDRs in this cannot yet be excluded [10], [57].

Besides their role in RNAi, several studies have described other (endogenous) functions of the RDRα (1, 2 and 6) genes [58], e.g. being involved in herbivore resistance (RDR1) [59], female gamete formation (RDR2 and 6) [60] or in developmental timing (RDR6) [61]. While a knockdown of RDR from the RDR1/2/6 class renders plants highly susceptible to many different viruses [11], their transcriptional up-regulation has been observed to lead to (elevated) resistance levels against different plant viruses [62].Viruses are able to counteract RNAi by coding for viral RSS proteins, and many of these have been shown to sequester siRNAs and prevent their uploading into RISC [63]. The presence of a viral RSS, however, does not seem to enable viruses to overcome elevated levels of resistance caused by transcriptional up-regulation of the RDR1/2/6 class of genes.

For RDR3, RDR4, and RDR5 a function has not yet been described [64]. How to explain the resistance mechanism of the *Ty-1*/*Ty-3* encoded RDRs remains speculative at present. The resistance spectrum of these alleles is not well studied; *Ty-3* also provides resistance to the bipartite *Tomato mottle virus* (ToMoV), but studies describing disease tests with other geminiviruses on *Ty-1*/*Ty-3* carrying lines are not available [21]. These genes act specifically on geminiviruses; what then is the identity of the (conserved?) Avr protein, and what are the characteristics of resistance breaking isolates? Considering the role of the DLDGD type of RDRs (1,2 and 6) in the generation of secondary siRNAs, irrespective of the RNA virus involved, it is tempting to propose a role of the DFDGD type of RDRs (3,4 and 5), and thus of *Ty-1*/*Ty-3*, in the formation of dsRNA too. Since *Ty-1*/*Ty-3* lines are resistant to TYLCV, but still allow for a symptomless (*Ty-1*) or an almost symptomless (*Ty-3*) infection with low titres of TYLCV, a resistance strategy as earlier described for the RDRα (1,2 and 6) genes could be possible, where transcriptional up regulation provides (elevated) resistance levels against different plant viruses.

In light of this, transcriptional expression analysis of *Ty-1* showed elevated expression levels in resistant lines compared to those in susceptible lines, even without TYLCV challenging. Whether differences in the *Ty-1* vs. *ty-1* protein or just those in transcriptional expression levels, or even a combination of both, are the cause of resistance remains to be investigated. However, since we did not observe hyper-susceptibility in tomato Moneymaker after silencing of the susceptible allele, as what is observed for Potato Virus X (PVX) and potato potyvirus Y (PVY) after silencing of *Nicotiana benthamiana RDR6* [14], a function of *ty-1* in resistance is highly unlikely. The functionality and transcriptional upregulation of *Ty-1* thus seems the most plausible reason to explain the resistance. To solve this issue, transgenic tomato lines (over)expressing either the resistant allele or the susceptible allele will be made. Analysis of the expression level and protein sequence of *Ty-1*/*ty-1* in other resistant/susceptible tomato varieties and wild species will additionally be informative and experiments for these are currently being prepared.

The observed resistance specificity of *Ty-1*/*Ty-3* against TYLCV does seem to contradict the idea that its transcriptional up regulation provides (elevated) resistance levels against other geminiviruses unless people have somehow overlooked a partial resistance to other, distinct geminiviruses. Furthermore, it is possible that the RDRγ (3, 4 and 5) class of genes may be involved in the generation of siRNAs that will mainly direct methylation of DNA and thereby support transcriptional silencing of geminivirus DNA genomes. If this hypothesis is true, this could explain why these genes will not confer (partial) resistance to most other plant viruses, of which ∼75% harbours an RNA genome and thus cannot be transcriptionally silenced by the siRNA-directed DNA methylation pathway.

The possibility of an alternate route for dsRNA formation during geminivirus infections, besides the one involving RDR1/2/6, is being supported by the observations that mutants lacking RDR1, RDR2 and RDR6 still revealed basal levels of RNA silencing and siRNA biogenesis, and plants infected with TYLCV only showed a moderate increase in susceptibility to geminiviruses in plants deficient in RDR2 and 6 [11], [47]. Whether the *Ty-1*/*Ty-3* encoded RDR represents a player in this, and how the resistance mechanism acts, will be a challenge to investigate in the near future.

## Materials and Methods

### Plant material

For fine-mapping *Ty-1* from *S. chilense* accession LA1969, a TYLCV-resistant commercial hybrid Tygress with an introgression between markers Mi23 and P6-25, reflecting the same interval as described by Verlaan et al. (2011), was used. This *Ty-1* introgression was done by Jaap Hoogstraten of the Royal Sluis Seed Company, and it is different from the LA1969 *Ty-1* introgression that was done in Israel [19]. This hybrid was self-pollinated to produce F_2_ progeny. Through two cycles of selection for recombination in this F_2_ population, two recombinants were identified and used to generate RILs by selfing and selection with marker genotyping for homozygous introgressions. The first recombination event resulted in a resistant RIL containing a *S. chilense* introgression flanked by markers Mi23 and HBa0045I03 and was used as a control (named as *Ty-1* RIL, Figure 1) in all *Ty-1* experiments. Another recombination event resulted in a resistant RIL containing a *S. chilense* introgression between markers T0774 and HBa0045I03. The susceptible Fla. 7776 was crossed to this inbred and an F_2_ population was generated. Approximately 2000 F_2_ plants were screened for recombination between the markers T0774 and SL_2.40ch06_30.891 and 13 recombinants were identified. These recombinants were selfed to develop F_4_ RILs as described before. RILs were evaluated, along with the controls Fla. 7776, Tygress and the *Ty-1* RIL in fall 2011. Four week-old seedlings were inoculated with TYLCV for 11 days then transplanted to the field on 4 October in a non-randomized trial with two replications of 4-plant plots. TYLCV disease severity was evaluated on each plant 6 weeks after exposure to whiteflies. For the three most informative recombinants (R7, R8 and R11) results were confirmed in the greenhouse using agroinoculation as described below.

Fla. 8680, which contains *Ty-3* within an approximately 27 cM introgression from the *S. chilense* accession LA2779, was crossed to the susceptible breeding line Fla. 7781 to produce an F_2_ population. F_2_ plants (n = 717) were individually screened in fall 2006 for recombination between the molecular markers C2_At2g39590 and T0834, located near the distal ends of the introgression. Recombinants selected from this F_2_ population were used to develop RILs as described above. The F_4_ and F_5_ RILs were evaluated for resistance in fall 2007 and spring 2008, respectively, in a randomized complete block design with three blocks and 12-plant plots. To further fine-map the *Ty-3* locus, three F_2_ sub-populations were developed using two key recombinants, i.e. 554 and 157 (Table S3). Sub-population A was an F_4_ generated by self-pollinating F_3_ progeny of recombinant 554 which were heterozygous for the introgression; sub-population B was an F_2_ derived from a cross between the susceptible breeding line Fla. 7776 and the F_5_ RIL of recombinant 554 (RIL 554). Sub-population C was also an F_2_ developed from a cross of Fla. 7776 and the F_5_ RIL of recombinant 157 (RIL 157). Seeds of all three sub-populations were sown and leaf tissue was collected from each plant at approximately 5 weeks after sowing. Sub-populations A and B were screened with the markers Mi23 and P6-25, and the markers T0774 and T0834 were used to screen sub-population C. Recombinants were transplanted to the field, along with controls, in early to mid-March, 2009. Controls included the TYLCV resistant commercial hybrids Tygress and SecuriTY 28, the resistant inbreds Fla. 8680 and Fla. 8602, the susceptible inbreds Horizon and Fla. 7776, RILs 554 and 157 and their F_1_ hybrids with Fla. 7776. One month after transplanting to the field, 6–8 cuttings were taken from each plant, rooted in a 1∶1 perlite, fine vermiculite media under mist for 2 weeks, then inoculated with whiteflies viruliferous for TYLCV for 11 days. Inoculated cuttings were transplanted to the field on 11 May in a non-randomized design with 3 replications of 2-plant plots, with the exception that only 2 replications were planted for recombinants having cross-overs outside the T0774 to P6-25 interval. TYLCV disease severity was evaluated on each plant at 5–6 weeks after exposure to whiteflies.

Self-pollinated seed was harvested from all original recombinant plants, and progeny were grown out in summer 2009 from 26 individuals with recombination between markers SL_2.40ch06_30.696 and cLEG-31-P16. Plants homozygous for the recombined introgression were selected for producing RILs. These RILs were grown in spring 2010, along with the controls Fla. 7776, Fla. 8680, the F_1_ hybrids between Fla. 7776 and each of RILs 554 and 157, and the commercial hybrid Tygress. Three week-old seedlings were inoculated with TYLCV for two weeks then transplanted to the field on 23 March in a randomized complete block design with three blocks and six-plant plots. TYLCV disease severity was evaluated on each plant at seven weeks after exposure to whiteflies.

### TYLCV inoculation and disease evaluation

Whitefly mediated inoculation: Plants were inoculated with whiteflies viruliferous for the TYLCV-IL strain according to the method of [65] with some modifications. Briefly, plants were exposed to viruliferous whiteflies in growth chambers for the specified period of time. After inoculation, the whiteflies were killed by treating plants with an insecticidal soap and with Admire (imidacloprid), and the plants were then transplanted to the field. Plants were rated for disease severity on a 0 to 4 disease severity index scale as described by Scott et al. (1996), where 0 = no symptoms and 4 = severe symptoms and stunting. Intermediate scores such as 1.5, 2.5, etc. were incorporated to allow for more precise disease severity ratings.

Agrobacterium mediated inoculation: An infectious TYLCV-IL clone (pTYCz40a) was used for agroinoculation using the method as described in [35]. Briefly, *A. tumefaciens* LBA4404 was transformed, cultured in LB, pelleted and resuspended in infiltration medium at an OD_600_ of 0.5. Three week old seedlings were infiltrated by pressure inoculation in the leaves with a needle-less syringe. For the VIGS experiments the agro infiltration was done two weeks after TRV inoculation.

### DNA extraction, molecular marker design and testing, and statistical analysis

DNA was extracted from young leaves using the cetyltrimethyl ammonium bromide (CTAB) protocol of [66] with minor modifications as described by [67]. Molecular markers used in this study were either publicly available, or were designed using the software Primer3 [68] from *Ty-3*-region BAC-end sequences, FOS-end sequences, the draft tomato genome available through the Sol Genomics Network (SGN; http://solgenomics.net/) [36], or from a private database of *S. lycopersicum* sequences. Polymerase chain reaction (PCR) parameters, primer sequences, restriction enzymes, and detection methods were described by [69] or [35]. Additional molecular markers designed are described in Table S5 and Figure S3, and used the same PCR parameters described by [69]. Analyses of variance, se calculations, and Duncan's multiple range tests were performed in SAS (Version 9.1; SAS Institute, Cary, NC). Mapping and interval analysis of *Ty-3* was performed in Windows QTL Cartographer 2.0 (2007, N.C. State University) using mean disease severity of the cuttings for each recombinant and a subset of molecular markers specific to the *Ty-3* region.

### Generation of TRV vectors for silencing

For gene silencing, the TRV based VIGS system as described in [70] was used. Briefly, fragments of approximately 350 base pairs of Solyc06g051160, Solyc06g051180 and Solyc06g051190 were amplified from *Ty-1* cDNA using primers compatible with the Gateway system (Table S6). After cloning to pENTR the inserts were sequenced to confirm their identity. Positive clones were selected for further processing of the inserts into the TRV2 vector and subsequently transformed to *Agrobacterium tumefaciens* strain GV3101.

### TRV infection by agrobacterium-mediated infiltration

A 3 ml culture of *A. tumefaciens* strain GV3101 containing the TRV replicons was grown overnight at 28°C, 200 RPM in appropriate selective LB medium. Cultures were transferred to 20 mL LB containing proper selection pressure, 10 mM MES and 200 µM acetosyringone, and further grown overnight in a 28°C shaker. *A. tumefaciens* cells were pelleted, and resuspended in infiltration buffer (20 g/L sucrose, 5 g/L MS salts (no vitamins), 10 mM MES) to a final OD_600_ of 1. Agro infiltration was performed on cotyledons of 10 day old seedlings using pressure inoculation with a 2, 5 mL syringe without a needle.

### Phylogenetic analysis

A neighbour joining tree with a bootstrap value of 1000 was generated using *MEGA* version 5 [71]. Arabidopsis RDR sequences were downloaded from The Arabidopsis Information Resource (www.arabidopsis.org) [72].

### Quantitative RT–PCR

For gene expression analysis, 17 day old seedlings were agroinoculated as described above. For the mock treatment infiltration buffer without bacteria was used. Top leaves of plants were harvested 0, 5, 12 and 19 days after TYLCV inoculation and grinded in liquid nitrogen using mortar and pestle. Total RNA was extracted by using the RNeasy Plant Mini Kit (Qiagen) as described by the manufacturer. One µg RNA was digested using DNase I (Amp. Grade) following the manufacturers protocol (Invitrogen) and cDNA was synthesized using the iScript cDNA Synthesis Kit following the protocol (Bio-Rad). Quantitative Real-Time PCR was performed in 10 µl reactions in a Bio-Rad iCycler iQ5 using SYBR Green Supermix (Bio-Rad) according to the protocol provided by the manufacturer.

For quantitative RT-PCR of *Ty-1*/*ty-1* the forward primer 180-F1 (5′-GGCAAAATATGCAGCCAGGCTTTCC-3′) and the reverse primer 180-R1 (5′-TCAGTATGTATACGAGGTTCGCCGT-3′) were used. As a reference the ACT gene was used as described by [73] with primers: ACT-F (5′-GAAATAGCATAAGATGGCAGACG-3′) and ACT-R (5′-ATACCCACCATCACACCAGTAT-3′). Gene expression levels were calculated using the ΔΔCt method as described by [74].

## Supporting Information

Figure S1 Interval mapping for TYLCV resistance on tomato chromosome 6. Maximal logarithm of odds (LOD) score for disease severity on cuttings from approximately 300 recombinant plants from the *Ty-3* fine mapping population. Approximate physical positions are based on the tomato genome assembly SL_2.40, available through the Sol Genomics Network (SGN; http://solgenomics.net/).
(PDF)

Figure S2 Determining the exact point of recombination in R7. Depicted is a part of the sequence of predicted gene Solyc06g051190, the first lines shows the spliced sequence, the second, third and fourth line show the genomic sequence of *ty-*1, *Ty-1* and R7 respectively. Based on the three SNPs that are present in this region the recombination point in R7 could be located in between the second and third SNP shown here.
(PDF)

Figure S3 SNPs of markers M17, M25, M27, M29, M31 in R7, R8 and R11. SNPs shown in black and grey were used to genotype R7, R8 and R11.
(PDF)

Figure S4 PCR strategy to prove that predicted Solyc06g051170, Solyc06g051180 and Solyc06g051190 are one gene. Primers used are indicated with their name (for primer sequences: Table S6), F4-R5 proved the connection between Solyc06g051180 and Solyc06g051190 and F6-R4 showed all three predicted genes are connected. F3-R10 was 1069 bp and F7-R7 was 786 bp, both as expected. F6-R4 had an expected size of 695 bp but the obtained fragment was 668 bp, for F4-R5 the expected size was 889 bp but the obtained fragment had a size of 925 bp. These size differences could be explained because the last predicted exon of Solyc06g051190 was not expressed and for Solyc06g051180 the first exon started earlier than predicted, the last exon was shorter than predicted. Finally for Solyc06g051170 the first predicted exon was not expressed.
(PDF)

Figure S5 Clustal W alignment of *A. thaliana* RDR1 to RDR6, *Ty-1*, *Ty-3* and *ty-1*. Differences between Ty-1, Ty-3 and MM are indicated with black boxes beneath the alignment.
(PDF)

Figure S6 Clustal W alignment of *A. thaliana* RDR3, RDR4, RDR5, *Ty-1*, *Ty-3* and *ty-1*. Differences between Ty-1, Ty-3 and MM are indicated with black boxes beneath the alignment.
(PDF)

Table S1 Agroinoculation disease test on the three most informative *Ty-1* RILS.
(PDF)

Table S2 Recombinant inbred lines (RILs) derived from the cross between tomato inbreds Fla. 7781 and Fla. 8680, their genotypes for the Ty-3 region of chromosome 6, and their phenotypes across two growing seasons.
(PDF)

Table S3 Tomato plants from the Ty-3 fine-mapping population selected for recombination in the Ty-3 region of chromosome 6, mean Tomato Yellow Leaf Curl Virus (TYLCV) disease severity of their cuttings, and their genotype throughout the region.
(PDF)

Table S4 Average Tomato yellow leaf curl virus disease severity index (DSI) for tomato cuttings evaluated in spring 2009.
(PDF)

Table S5 Molecular markers on chromosome 6 of tomato.
(PDF)

Table S6 Primers used in this study.
(PDF)
