# Supplementary figures and images for: The Tomato Yellow Leaf Curl Virus Resistance Genes Ty-1 and Ty-3 Are Allelic and Code for DFDGD-Class RNA–Dependent RNA Polymerases
Source: PLoS Genet. 2013 Mar 28;9(3):e1003399. doi: 10.1371/journal.pgen.1003399 (PMC3610679; doi:10.1371/journal.pgen.1003399)

Figure S1: Interval mapping for TYLCV resistance on tomato chromosome 6.

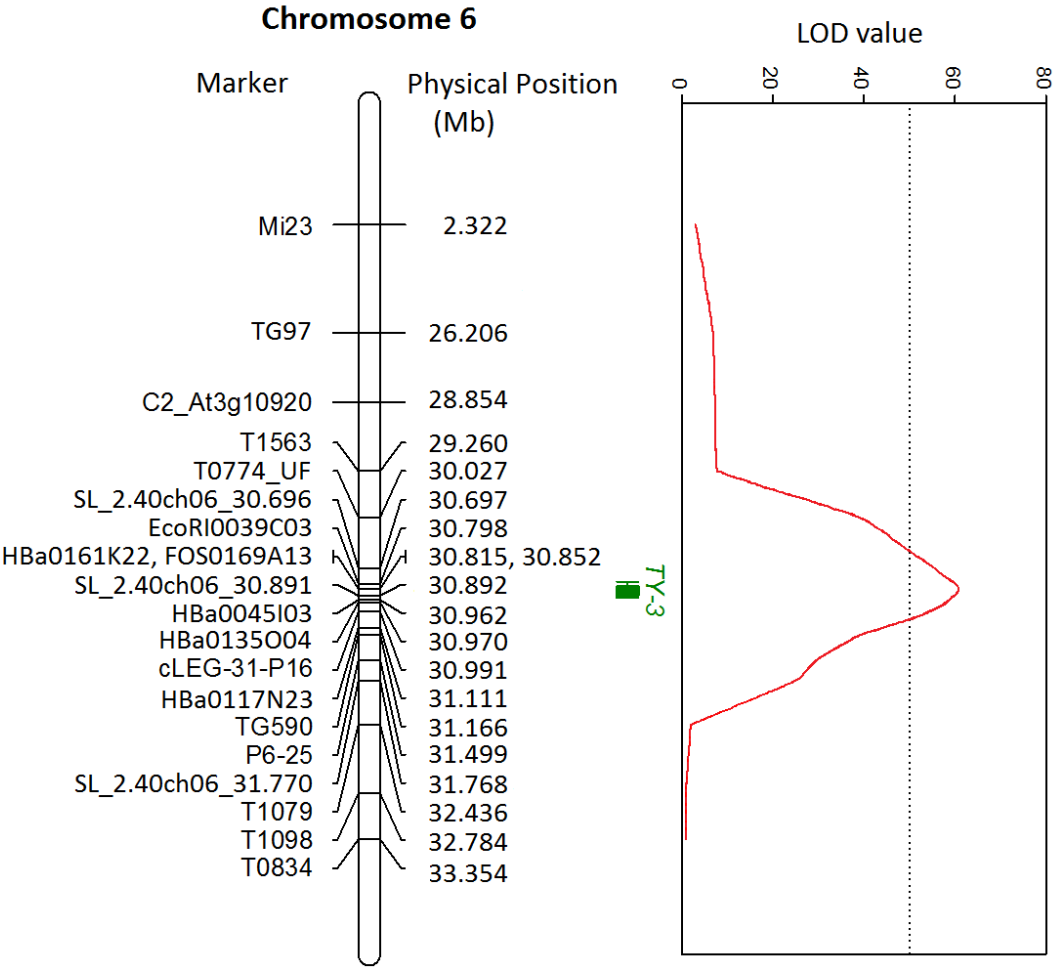

Supplement: Figure S1 — Interval mapping for TYLCV resistance on tomato chromosome 6. Maximal logarithm of odds (LOD) score for disease severity on cuttings from approximately 300 recombinant plants from the Ty-3 fine mapping population. Approximate physical positions are based on the tomato genome assembly SL_2.40, available through the Sol Genomics Network (SGN; http://solgenomics.net/). (PDF) [file pgen.1003399.s001.pdf]

**Figure S2: Determining the exact point of recombination in R7.**

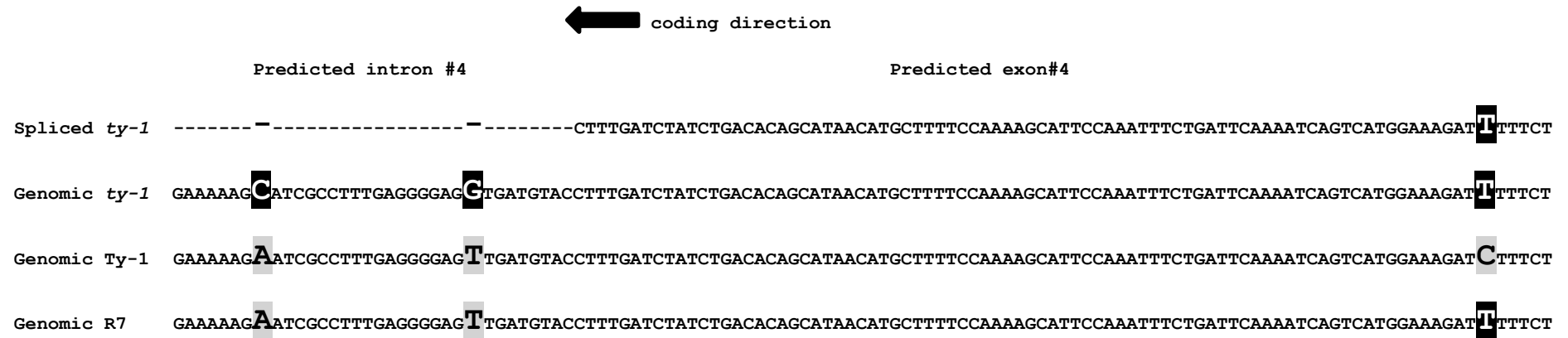

Supplement: Figure S2 — Determining the exact point of recombination in R7. Depicted is a part of the sequence of predicted gene Solyc06g051190, the first lines shows the spliced sequence, the second, third and fourth line show the genomic sequence of ty-1, Ty-1 and R7 respectively. Based on the three SNPs that are present in this region the recombination point in R7 could be located in between the second and third SNP shown here. (PDF) [file pgen.1003399.s002.pdf]

Figure S6. Clustal W alignment of *A. thaliana* RDR3, RDR4, RDR5, Ty-1, Ty-3 and ty-1

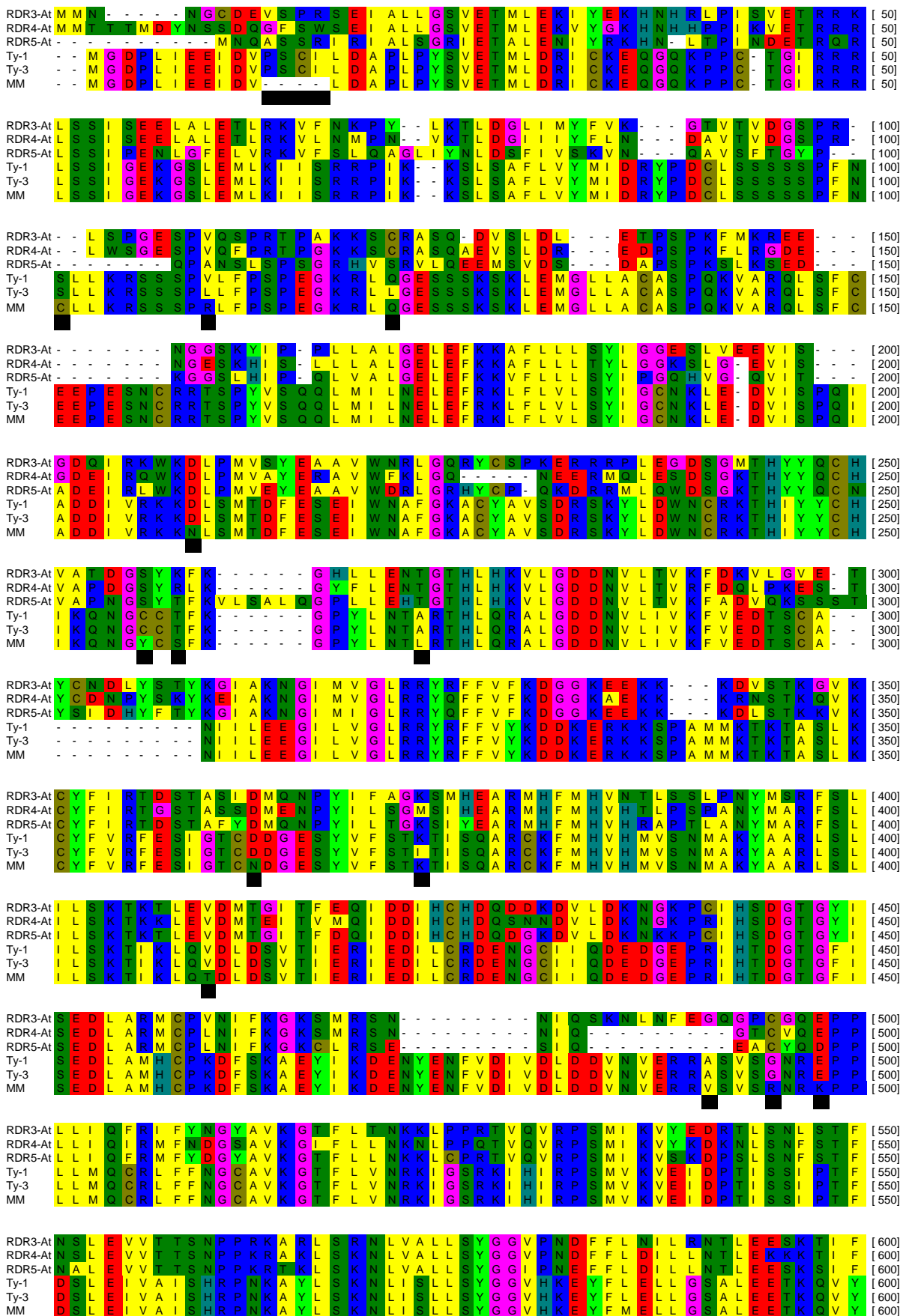

Supplement: Figure S6 — Clustal W alignment of A. thaliana RDR3, RDR4, RDR5, Ty-1, Ty-3 and ty-1. Differences between Ty-1, Ty-3 and MM are indicated with black boxes beneath the alignment. (PDF) [file pgen.1003399.s006.pdf]
