# Supplementary material for: The Tomato Yellow Leaf Curl Virus Resistance Genes Ty-1 and Ty-3 Are Allelic and Code for DFDGD-Class RNA–Dependent RNA Polymerases
Source: PLoS Genet. 2013 Mar 28;9(3):e1003399. doi: 10.1371/journal.pgen.1003399 (PMC3610679; doi:10.1371/journal.pgen.1003399)
Supplement: Figure S3 — SNPs of markers M17, M25, M27, M29, M31 in R7, R8 and R11. SNPs shown in black and grey were used to genotype R7, R8 and R11. (PDF) [file pgen.1003399.s003.pdf]

Figure S3: SNPs of markers M17, M25, M27, M29, M31 in R7, R8 and R11.

```
M17
ty-1 TTCTCTCAATCGGACAATCC
Ty-1 TTCTCCTAATCAAACAATCC
R7   TTCTCCTAATCAAACAATCC
R8   TTCTCTCAATCGGACAATCC
R11  TTCTCTCAATCGGACAATCC

M25
ty-1 TACACAACATTGAAACTGTAAATCCGTAC
Ty-1 TACACGACATTGAAACTGTAAATCTGTAC
R7   TACACGACATTGAAACTGTAAATCTGTAC
R8   TACACAACATTGAAACTGTAAATCCGTAC
R11  TACACAACATTGAAACTGTAAATCCGTAC

M27
ty-1 TAATGTCAAAATTGATGGTCA
Ty-1 TAATGCCAAAATTGACGGTCA
R7   TAATGCCAAAATTGACGGTCA
R8   TAATGTCAAAATTGATGGTCA
R11  TAATGTCAAAATTGATGGTCA

M29
ty-1 TAGTTCAATGACCTCTTATGAT
Ty-1 TAGTTCACTGACCTCTTAAGAT
R7   TAGTTCAATGACCTCTTATGAT
R8   TAGTTCAATGACCTCTTATGAT
R11  TAGTTCAATGACCTCTTATGAT

M31
ty-1 GAGAAAGGTAACAGCAACATT
Ty-1 GAGAAAGGGAACAGTAACATT
R7   GAGAAAGGTAACAGCAACATT
R8   GAGAAAGGTAACAGCAACATT
R11  GAGAAAGGGAACAGTAACATT
```
