# Supplementary material for: The Tomato Yellow Leaf Curl Virus Resistance Genes Ty-1 and Ty-3 Are Allelic and Code for DFDGD-Class RNA–Dependent RNA Polymerases
Source: PLoS Genet. 2013 Mar 28;9(3):e1003399. doi: 10.1371/journal.pgen.1003399 (PMC3610679; doi:10.1371/journal.pgen.1003399)

Figure S4: PCR strategy to prove that predicted genes Solyc06g051170, Solyc06g051180 and Solyc06g051190 together code for one gene

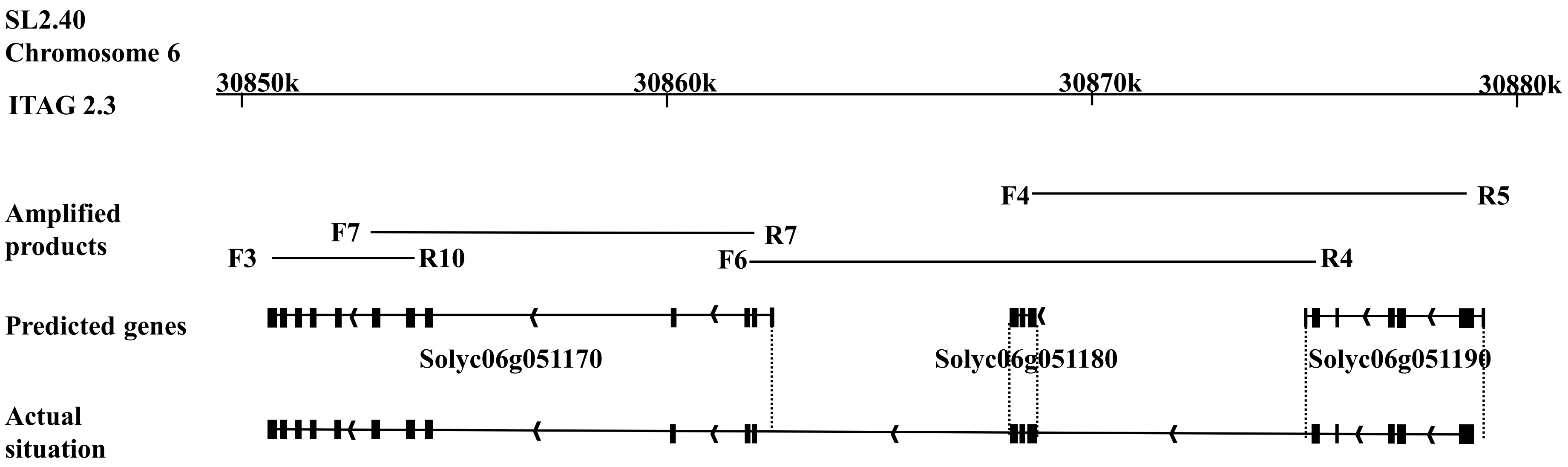

Supplement: Figure S4 — PCR strategy to prove that predicted Solyc06g051170, Solyc06g051180 and Solyc06g051190 are one gene. Primers used are indicated with their name (for primer sequences: Table S6), F4-R5 proved the connection between Solyc06g051180 and Solyc06g051190 and F6-R4 showed all three predicted genes are connected. F3-R10 was 1069 bp and F7-R7 was 786 bp, both as expected. F6-R4 had an expected size of 695 bp but the obtained fragment was 668 bp, for F4-R5 the expected size was 889 bp but the obtained fragment had a size of 925 bp. These size differences could be explained because the last predicted exon of Solyc06g051190 was not expressed and for Solyc06g051180 the first exon started earlier than predicted, the last exon was shorter than predicted. Finally for Solyc06g051170 the first predicted exon was not expressed. (PDF) [file pgen.1003399.s004.pdf]
