# Supplementary material for: The Tomato Yellow Leaf Curl Virus Resistance Genes Ty-1 and Ty-3 Are Allelic and Code for DFDGD-Class RNA–Dependent RNA Polymerases
Source: PLoS Genet. 2013 Mar 28;9(3):e1003399. doi: 10.1371/journal.pgen.1003399 (PMC3610679; doi:10.1371/journal.pgen.1003399)
Supplement: Figure S5 — Clustal W alignment of A. thaliana RDR1 to RDR6, Ty-1, Ty-3 and ty-1. Differences between Ty-1, Ty-3 and MM are indicated with black boxes beneath the alignment. (PDF) [file pgen.1003399.s005.pdf]

Figure S5. Clustal W alignment of *A. thaliana* RDR1 to RDR6, *Ty-1* , *Ty-3* and *ty-1*.

```
RDR1-At  ----------MGKTIQVFGFPNGVSAEEVKKFLERLTGSGTVYAIKVRQP  [ 50]
RDR2-At  ---MVSETTTNRSTVKISNVPQTIVADELLRFLELHLGEDTVFALEIPTT  [ 50]
RDR6-At  MGSEGNMKKSVVTQVSIGGFGESTTAKQLTDYLEDEVGIVWRCRLKTSWT  [ 50]
RDR3-At  ---------------MMN-----NGCDEVSPRSEIALLGSVETMLEKIYE  [ 50]
RDR4-At  ---------------MMTTTMDYNSSDQGFSWSEIALLGSVETMLEKVYG  [ 50]
RDR5-At  -------------------------MNQASSRIRIALSGRIETALENIYR  [ 50]
Ty-1     -----------------MGDPLIEEIDVPSCILDAPLPYSVETMLDRICK  [ 50]
Ty-3     -----------------MGDPLIEEIDVPSCILDAPLPYSVETMLDRICK  [ 50]
MM       -----------------MGDPLIEEIDV----LDAPLPYSVETMLDRICK  [ 50]

RDR1-At  KKGG--------------------PRVYAIVQFTS-ERHTRLIITAAAER  [100]
RDR2-At  RDNWK-------------------PRDFARVQFTTLEVKSRAQLLSSQSK  [100]
RDR6-At  PPGSYPNFEIADTSNIPSIDEYKKVEPHAFVHFAVFESAGRAMDAAGQCN  [100]
RDR3-At  KHNH----------------------------RLPISVETRRKLSSISEE  [100]
RDR4-At  KHNH----------------------------HPPIKVETRRRLSSISEE  [100]
RDR5-At  KHN-----------------------------LTPINDETRQRLSSIPEN  [100]
Ty-1     EQG-----------------------------QKPPCTGIRRRLSSIGEK  [100]
Ty-3     EQG-----------------------------QKPPCTGIRRRLSSIGEK  [100]
MM       EQG-----------------------------QKPPCTGIRRRLSSIGEK  [100]

RDR1-At  LYYGRSYLKAFEVEQD----IVPKPRASLHTISGLKMFFGCQVS-TKKFL  [150]
RDR2-At  LLFKTHNLRLSEAYDD----IIPRPVDPRKRLDDIVLTVGFPESDEKRFC  [150]
RDR6-At  LILDGQPLKVSLGPKNPYSLNQRRRTTVPYKLAGITLEIGTLVS-RDDFF  [150]
RDR3-At  LALETLRKVFNKPY--------------LKTLDGLIMYFVK---------  [150]
RDR4-At  LALETLRKVLNMPN--------------VKTLDGIIIYFLN---------  [150]
RDR5-At  LGFELVRKVFSLQAGL------------IYNLDSFIVSKVN---------  [150]
Ty-1     GSLEMLKIISRRPIK--------------KSLSAFLVYMIDRYP------  [150]
Ty-3     GSLEMLKIISRRPIK--------------KSLSAFLVYMIDRYP------  [150]
MM       GSLEMLKIISRRPIK--------------KSLSAFLVYMIDRYP------  [150]

RDR1-At  TLWSAQDVCVSFGIGMRKLHFSFSWYQKDYRLELSYENIWQIDLHSPQGR  [200]
RDR2-At  ALEKWDGVRCWILTEKRRVEFWVWESGDCYKIEVRFEDIIETLSCCVNGD  [200]
RDR6-At  VSWRAEGVDFLVDPFDNTCKFCFRKSTAFSFKDAVMHAVINCDYKLELLV  [200]
RDR3-At  ------------------------------------GTVTVDGSPR---L  [200]
RDR4-At  ------------------------------------DAVTVDGSPR---L  [200]
RDR5-At  ------------------------------------QAVSFTGYP-----  [200]
Ty-1     ------------------------------------DCLSSSSSPFNSLL  [200]
Ty-3     ------------------------------------DCLSSSSSPFNSLL  [200]
MM       ------------------------------------DCLSSSSSPFNCLL  [200]

RDR1-At  SSKF--LVIQVIGAPKIFEKEDQPINLLFG--IMDFYSDGS------DEQ  [250]
RDR2-At  ASEIDAFLLKLKYGPKVFKRVTVHIATKFKSDRYRFCKEDF------DFM  [250]
RDR6-At  RDIQTVRQYKTLHGFVLILQLASSPRVWYRTADDDIYDTVPGDLLDDDDP  [250]
RDR3-At  SPGESPVQSPRTPAKKSCRASQ-DVSLDL---ETPSPK------------  [250]
RDR4-At  WSGESPVQFPRTPGKKSCRASQAEVSLDR---EDPSPK------------  [250]
RDR5-At  ----QPANSLSPSGRHVSRVLQEEMSVDS---DAPSPK------------  [250]
Ty-1     KRSSSPVLFPSPEGKRLQGESSSKSKLEMGLLACASPQKVA------RQL  [250]
Ty-3     KRSSSPLLFPSPEGKRLLGESSSKSKLEMGLLACASPQKVA------RQL  [250]
MM       KRSSSPRLFPSPEGKRLQGESSSKSKLEMGLLACASPQKVA------RQL  [250]

RDR1-At  WIRTTDFTSSSCIGQSTAFCLELPVHLNVPDFRENFANYAEHRASSFLIE  [300]
RDR2-At  WIRTTDFSGSKSIGTSTCFCLEVHNGSTMLDIFSGLPYYREDTLSLTYVD  [300]
RDR6-At  WIRTTDFTQVGAIGRCHSYRVLISPRYEN-KLRTALDYFRMRRVQEERVR  [300]
RDR3-At  FMKREENGGSKYIP-----PLLALGELEFKKAFLLLSYIGGESLVEEVIS  [300]
RDR4-At  FLRGDENGESKHIS-----LLLALGELEFKKAFLLLTYLGGKSLG-EVIS  [300]
RDR5-At  SLKSEDKGGSLHIP-----QLVALGELEFKKVFLLLSYIPGQHVG-QVIT  [300]
Ty-1     SFCEEPESNCRRTSPYVSQQLMILNELEFRKLFLVLSYIGCNKLE-DVIS  [300]
Ty-3     SFCEEPESNCRRTSPYVSQQLMILNELEFRKLFLVLSYIGCNKLE-DVIS  [300]
MM       SFCEEPESNCRRTSPYVSQQLMILNELEFRKLFLVLSYIGCNKLE-DVIS  [300]

RDR1-At  SGSSYSSNANTLVPVVDPPPGFSLPFEILFKLNTLVQNACLSG-PALDLD  [350]
RDR2-At  -GKTFASAAQIVPLLNAAILGLEFPYEILFQLNALVHAQKISLFAASDME  [350]
RDR6-At  WPPRIRNEPCFGEPVSDHFFCIHHKEGISFEIMFLVNSVLHRGVFNQFQL  [350]
RDR3-At  ---GDQIRKWKDLPMVSYEAAVWNRLGQRYCSPKERRRPLEGDSGMTHYY  [350]
RDR4-At  ---GDEIRQWKDLPMVAYERAVWFKLGQ-----NEERMQLESDSGKTHYY  [350]
RDR5-At  ---ADEIRLWKDLPMVEYEAAVWDRLGRHYCP-QKDRRMLQWDSGKTHYY  [350]
Ty-1     PQIADDIVRKKDLSMTDFESEIWNAFGKACYAVSDRSKYLDWNCRKTHIY  [350]
Ty-3     PQIADDIVRKKDLSMTDFESEIWNAFGKACYAVSDRSKYLDWNCRKTHIY  [350]
MM       PQIADDIVRKKNLSMTDFESEIWNAFGKACYAVSDRSKYLDWNCRKTHIY  [350]

RDR1-At  FYRLLNQKKYDRALIDHCLEKLFHLGECCYEPAHWLRDEYKKWISKGK-L  [400]
RDR2-At  LIKILRGMSLETALV--ILKKLHQQSSICYDPVFFVKTQMQSVVKKMKHS  [400]
RDR6-At  TERFFDLLRNQPKDVN---IASLKHLCTYKRPVFDAYKRLKLVQEWIQKN  [400]
RDR3-At  QCHVATDGSYKFK------GHLLENTGTHLHKVLGDDNVLTVKFDKVLGV  [400]
RDR4-At  QCHVAPDGSYRLK------GYFLENTGTHLHKVLGDDNVLTVRFDQLPKE  [400]
RDR5-At  QCNVAPNGSYTFKVLSALQGPLLEHTGTHLHKVLGDDNVLTVKFADVQKS  [400]
Ty-1     YCHIKQNGCCTFK------GPYLNTARTHLQRALGDDNVLIVKFVED---  [400]
Ty-3     YCHIKQNGCCTFK------GPYLNTARTHLQRALGDDNVLIVKFVED---  [400]
MM       YCHIKQNGYCSFK------GPYLNTLRTHLQRALGDDNVLIVKFVED---  [400]

RDR1-At  PLSPTISLDDG-LVYMYRVQVTPARVYFSGPEVNVSNRVLRH-YSKYINN  [450]
RDR2-At  PASAYKRLTEQNIMSCQRAYVTPSKIYLLGPELETANYVVKN-FAEHVSD  [450]
RDR6-At  PKLLGSHEQSEDISEIRRLVITPTRAYCLPPEVELSNRVLRR-YKAVAER  [450]
RDR3-At  E-TYCNDLYSTYKGIAKNGIMVGLRRYRFFVFKDGGKEEKK---KDVSTK  [450]
RDR4-At  S-TYCDNPYSKYKEIAKNGIMVGLRRYQFFVFKDGGKAEKK---KRNSTK  [450]
RDR5-At  SSTYSIDHYFTYKGIAKNGIMIGLRRYQFFVFKDGGKEEKK---KDLSTK  [450]
Ty-1     --TSCAN------IILEEGILVGLRRYRFFVYKDDKERKKSPAMMKTKTA  [450]
Ty-3     --TSCAN------IILEEGILVGLRRYRFFVYKDDKERKKSPAMMKTKTA  [450]
MM       --TSCAN------IILEEGILVGLRRYRFFVYKDDKERKKSPAMMKTKTA  [450]
```

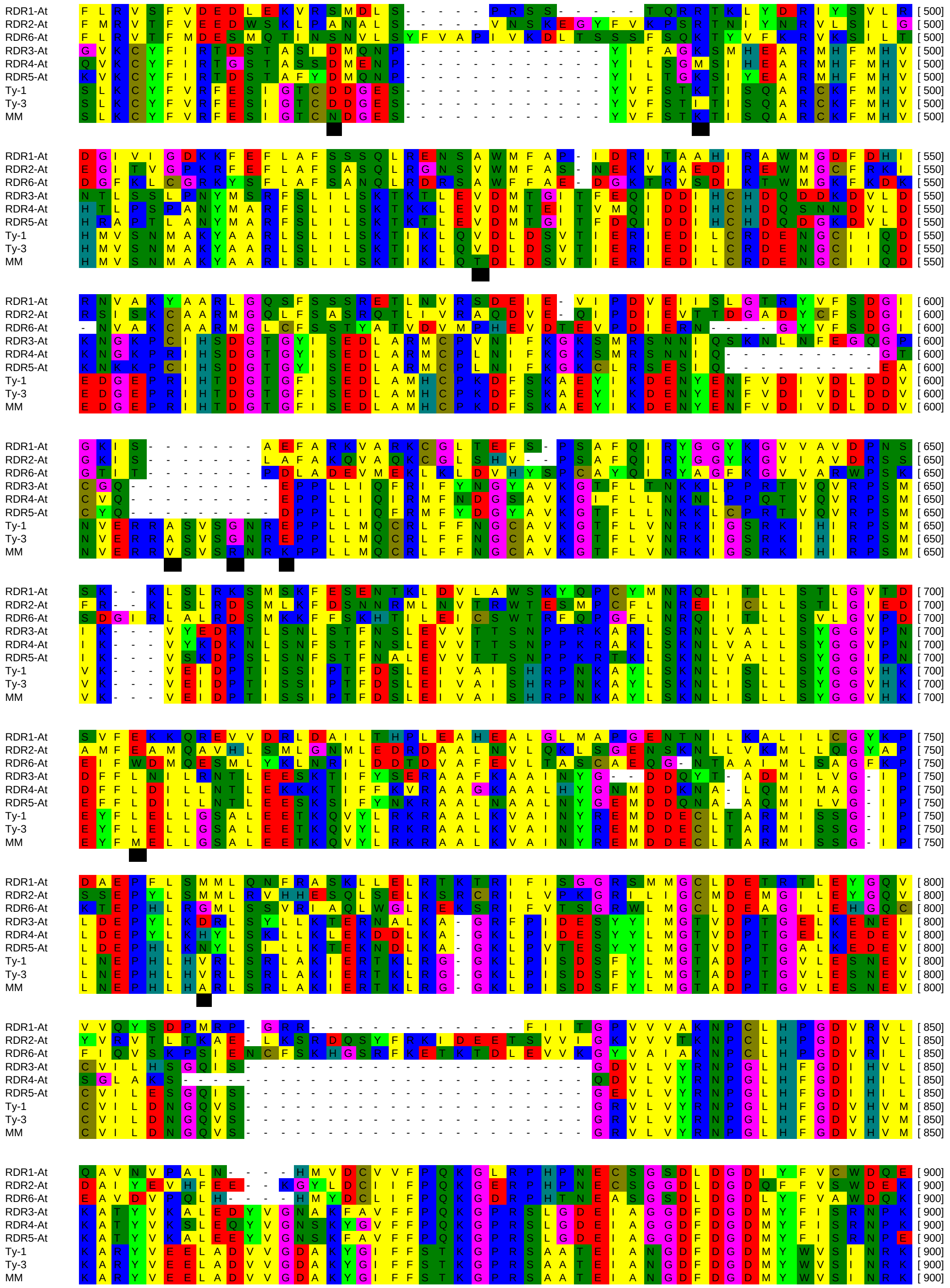

```
RDR1-At  LVPP--RTSEPMDYTPE-------PTQILDHDVTIEEVEEYFANY-----  [ 950]
RDR2-At  IIPS--EMDPPMDYAGS-------RPRLMDHDVTLEEIHKFFVDY-----  [ 950]
RDR6-At  LIPPNRKSYPAMHYDAA-------EEKSLGRAVNHQDIIDFFARN-----  [ 950]
RDR3-At  LLEHFKPSEPWVSSSKPSKIYCGRKPSELSEEELEEELFKMFLKARFCKR  [ 950]
RDR4-At  LLEHYKPSEPWVSSSPRSKIYTGRQPSELSPEQLEEELFKIFLKTGFSPS  [ 950]
RDR5-At  LLENFKPSEPWVSLTPPSKSNSGRAPSQLSPEELEEELFEMFLTAGFHAS  [ 950]
Ty-1     LVDSYTTSRPWIRMHSTPKAVS-KKPSEFSADELEYELFRQFLEAKSKGA  [ 950]
Ty-3     LVDSYTTSRPWIRMHSTPKAVS-KKPSEFSADELEYELFRQFLEAKSKGA  [ 950]
MM       LVDSYTTSRPWIRMHSTPNAVS-KKPSEFSADELEYELFRQFLEAKSKGA  [ 950]

RDR1-At  ----IVNDSLGIIANAHTAFADK--EPLKAFSDPCIELAKKFSTAVDFPK  [1000]
RDR2-At  ----MISDTLGVISTAHLVHADR--DPEKARSQKCLELANLHSRAVDFAK  [1000]
RDR6-At  ----LANEQLGTICNAHVVHADR--SEYGAMDEECLLLAELAATAVDFPK  [1000]
RDR3-At  DVIGMAADCWLGIMDPFLTLGDESAKEKYERKKNILKLIDIYYDALDAPK  [1000]
RDR4-At  SVIGQAADSWLAIMDRFLTLGDENVKEKAEMKKKMLKLTDIYYDAIDAPK  [1000]
RDR5-At  NVIGIAADSWLTIMDRFLILGDDRAEEKAEMKKKMLELIDIYYDALDAPK  [1000]
Ty-1     NMS-LAADSWLAFMDRLLMLRDDNVDDMHSLKGKMLHLIDIYYDALDAPK  [1000]
Ty-3     NMS-LAADSWLAFMDRLLMLRDDNVDDMHSLKGKMLHLIDIYYDALDAPK  [1000]
MM       NMS-LAADSWLAFMDRLLTLRDDNVDDMHSLKGKMLHLIDIYYDALDAPK  [1000]

RDR1-At  TGVAAVIPQHLYVKEYPDFMEKPDKPTYESKNVIGKLFREVKERAPPLIS  [1050]
RDR2-At  TGAPAEMPYALKPREFPDFLERFEKPTYISESVFGKLYRAVKS---SLAQ  [1050]
RDR6-At  TGKIVSMPFHLKPKLYPDFMGKEDYQTYKSNKILGRLYRRVKEVYDEDAE  [1050]
RDR3-At  KGAKVDLPPDLEIKNFPHYMERDPKRDFRSTSILGLIFDTVDS------H  [1050]
RDR4-At  TGTEVNLPLDVKVDLFPHYMERN--KTFKSTSILGLIFDTVDF------H  [1050]
RDR5-At  KGDKVYLPNKLKPDIFPHYMERD--KKFQSTSILGLIFDFVKS------Q  [1050]
Ty-1     SGKKVSIPHYLKANKFPHYMEKGNSCSYHSTSILGQIYDHVDS------Y  [1050]
Ty-3     SGKKVSIPHYLKANKFPHYMEKGNSCSYHSTSILGQIYDHVDS------Y  [1050]
MM       SGKKVSIPHYLKANKFPHYMEKGNSCSYHSTSILGQIYDHVDS------Y  [1050]

RDR1-At  IKSF-TLDVASKSYDKDMEVDGFEEYVDEAFYQKANYDFKLGNLMDYYGI  [1100]
RDR2-At  RKPE-AESEDTVAYDVTLEEAGFESFIETAKAHRDMYGEKLTSLMIYYGA  [1100]
RDR6-At  ASSEESTDPSAIPYDAVLEIPGFEDLIPEAWGHKCLYDGQLIGLLGQYKV  [1100]
RDR3-At  NAEEPPPS-EISKLWYFEDEPVPKSHMDKFTSWYENYRSEMSQAMMETDK  [1100]
RDR4-At  NAEDTTPS-GISKLQCFEDEPVSEFDMEKCKLWHKDYRKEMCQAMNSDD-  [1100]
RDR5-At  TTEEPSPSSEISKLPCFEDEPVSEFHMQKCRLWYDNYRTEMTQAMKTDK-  [1100]
Ty-1     PDEDLCIT-EISKLPCFEVE-IPQRCMTLWRGRYEEYKKDMTQAMNLDCE  [1100]
Ty-3     PDEDLCIT-EISKLPCFEVE-IPQRCMTLWRGRYEEYKKDMTQAMNLDCE  [1100]
MM       PDEDLCIT-EISKLPCFEVE-IPQRCMTLWRGRYEEYKKDMTRAMNFDCE  [1100]

RDR1-At  KTEAEILSGGIMRMSKSFTKRRD------AESIGRAVRALRKETLSLFNA  [1150]
RDR2-At  ANEEEILTG-ILKTKEMYLARDNRRYGDMKDRITLSVKDLHKEAMGWFEK  [1150]
RDR6-At  QKEEEIVTGHIWSMPKYTSKKQG----ELKERLKHSYNSLKKEFRKVFEE  [1150]
RDR3-At  VKRNQLTNEVIQRYKQDFYGAAG------------------------FED  [1150]
RDR4-At  ---DDSCNEVIQKYKQEFYSAAG------------------------FKE  [1150]
RDR5-At  ---DESANEVIQRYKQEFYGAAG------------------------FED  [1150]
Ty-1     LR-ITSCNEVIKKYKMLLYGAVE------------------------FEQ  [1150]
Ty-3     LR-ITPCNEVIKKYKMLLYGAVE------------------------FEQ  [1150]
MM       LR-ITSCNEVIKKYKMLLYGAVE------------------------FEQ  [1150]

RDR1-At  S-EEEENES---------AKASAWYHVTYHSSYWGL-----YNEGLNRDH  [1200]
RDR2-At  SCEDEQQKK---------KLASAWYYVTYNPNHR--------DEKLT---  [1200]
RDR6-At  TIPDHENLSEEEKNILYEKKASAWYHVTYHPEWVKKSLELQDPDESSHAA  [1200]
RDR3-At  SNKSLEELYP---------QALALYNVVYDYA-IQEGVAK----------  [1200]
RDR4-At  SKKILEELYP---------KALALYNVT----------------------  [1200]
RDR5-At  SKKSLEELYP---------QALALYKIVYDYA-IHAGVSK----------  [1200]
Ty-1     TVRKTEDIFD---------EALAIYHVTYDNARITYSIEK----------  [1200]
Ty-3     TVRKTEDIFD---------EALAIYHVTYDNARITYSIEK----------  [1200]
MM       TVRKTEDIFD---------EALAIYHVTYDNARITYSIEK----------  [1200]

RDR1-At  FLSFAWCVYDKLVRIKKTN--LGRRQRQETLERLDHVLRFG-  [1242]
RDR2-At  FLSFPWIVGDVLLDIKAEN--AQRQSVEEKTSGLVSI-----  [1242]
RDR6-At  MLSFAWIAADYLARIKIRSREMGSIDSAKPVDSLAKFLAQRL  [1242]
RDR3-At  -CTFAWNVAGPVLCKFYL-KKTKDKSVVASTSVLKKLLG---  [1242]
RDR4-At  ------------------------------------------  [1242]
RDR5-At  -CRFVWKVAGPVLCRFYLNKKMQEKCLVCAPSVLKELWG---  [1242]
Ty-1     -CGFAWKVAGSALCRIHAMYRKE-KDLPILPSVLQEIL*---  [1242]
Ty-3     -CGFAWKVAGSALCRIHAMYRKE-KDLPILPSVLQEIL*---  [1242]
MM       -CGFAWKVAGSALCRIHAMYRKE-KDLPILPSVLQEIL*---  [1242]
```
