# Supplementary material for: The Tomato Yellow Leaf Curl Virus Resistance Genes Ty-1 and Ty-3 Are Allelic and Code for DFDGD-Class RNA–Dependent RNA Polymerases
Source: PLoS Genet. 2013 Mar 28;9(3):e1003399. doi: 10.1371/journal.pgen.1003399 (PMC3610679; doi:10.1371/journal.pgen.1003399)
Supplement: Table S1 — Agroinoculation disease test on the three most informative Ty-1 RILS. (PDF) [file pgen.1003399.s007.pdf]

Supplemental table 1: Agroinoculation disease test on the three most informative *Ty-1* RILS

Line	Average DI[1]	N[2]	Markers[3]									
			M-H304P16	M-H309K01	T1563	T0774	MSc05732-3	MSc05732-4	SL_2.40ch06_30.696	HBa0161K22	FOS169A13	SL_2.40ch06_30.891
R7	1.3 ± 0.5	7	-	-	-	+	+	+	+	+	+	-
	0	7	-	-	-	+	+	+	+	+	+	/
	0	3	-	-	-	+	+	+	+	+	+	+
R8	4	6	-	-	-	+	+	+	+	+	-	-
	4	10	-	-	-	/	/	/	/	/	-	-
	4	6	-	-	-	-	-	-	-	-	-	-
R11	4	3	-	-	-	-	-	-	-	-	-	+
	4	6	-	-	-	-	-	-	-	-	-	/
	4	1	-	-	-	-	-	-	-	-	-	-
R12	0	4	-	-	-	-	-	-	+	+	+	+
	0	5	-	-	-	-	-	-	/	/	/	/
	4	2	-	-	-	-	-	-	-	-	-	-

[1] Average Disease Index (DI)

[2] Numbers of plant tested

[3] + = homozygous *Solanum chilense* , / = heterozygous, - = homozygous *S. lycopersicum*
