# Supplementary material for: The Tomato Yellow Leaf Curl Virus Resistance Genes Ty-1 and Ty-3 Are Allelic and Code for DFDGD-Class RNA–Dependent RNA Polymerases
Source: PLoS Genet. 2013 Mar 28;9(3):e1003399. doi: 10.1371/journal.pgen.1003399 (PMC3610679; doi:10.1371/journal.pgen.1003399)
Supplement: Table S2 — Recombinant inbred lines (RILs) derived from the cross between tomato inbreds Fla. 7781 and Fla. 8680, their genotypes for the Ty-3 region of chromosome 6, and their phenotypes across two growing seasons. (PDF) [file pgen.1003399.s008.pdf]

Supplemental table 2. Recombinant inbred lines (RILs) derived from the cross between tomato inbreds Fla. 7781 and Fla. 8680, their genotypes for the *Ty-3* region of chromosome 6, and their phenotypes across two growing seasons.

	Chromosome 6 Introgressed Segment[Z]												Mean RIL Rating[Y]	
Recombinant	C2_At2g39590	Mi23	TG436	TG178	T0892	T1563	**T0774**	cLEG-31-P16	**P6-25**	T1079	T1098	T0834	Fall 2007	Spring 2008
683	+	+	-	-	-	-	-	-	-	-	-	-		3.4^{ab}
343	+	+	+	-	-	-	-	-	-	-	-	-	$1.8^{\text{f-i}}$	$2.5^{\text{c-e}}$
463	+	+	+	-	-	-	-	-	-	-	-	-		3.3^{ab}
11	+	+	+	+	-	-	-	-	-	-	-	-	3.0^{b}	3.7^{ab}
359	+	+	+	+	-	-	-	-	-	-	-	-	3.2^{ab}	3.9^{ab}
503	+	+	+	+	-	-	-	-	-	-	-	-	$1.2^{\text{k-n}}$	3.0^{bc}
506	+	+	+	+	+	+	-	-	-	-	-	-	2.4^{cd}	3.0^{bc}
724	+	+	+	+	+	+	-	-	-	-	-	-	2.3^{de}	$2.7^{\text{c-e}}$
186	+	+	+	+	+	+	+	-	-	-	-	-	3.0^{b}	3.7^{ab}
403	+	+	+	+	+	+	+	-	-	-	-	-	$2.0^{\text{d-g}}$	$2.1^{\text{e-h}}$
554	+	+	+	+	+	+	+	+	+	-	-	-	$2.1^{\text{d-f}}$	$0.9^{\text{o-r}}$
78	+	+	+	+	+	+	+	+	+	+	-	-	$1.3^{\text{j-m}}$	$1.0^{\text{m-r}}$
705	+	+	+	+	+	+	+	+	+	+	-	-	$1.1^{\text{k-n}}$	$2.1^{\text{e-h}}$
432	+	+	+	+	+	+	+	+	+	+	+	-	$0.8^{\text{n-p}}$	$1.6^{\text{h-n}}$
362	-	+	+	+	+	+	+	+	+	+	+	+	$0.6^{\text{o-q}}$	$1.6^{\text{h-m}}$
116	-	+	+	+	+	+	+	+	+	+	+	+	$0.6^{\text{o-r}}$	$0.9^{\text{o-r}}$
718	-	-	-	-	+	+	+	+	+	+	+	+	$1.3^{\text{j-l}}$	$1.9^{\text{f-j}}$
688	-	-	-	-	+	+	+	+	+	+	+	+	$1.1^{\text{l-m}}$	$0.9^{\text{o-r}}$
552	-	-	-	-	+	+	+	+	+	+	+	+	$1.2^{\text{k-n}}$	$2.0^{\text{f-i}}$
71	-	-	-	-	+	+	+	+	+	+	+	+		$1.5^{\text{h-o}}$
719	-	-	-	-	-	+	+	+	+	+	+	+	$1.0^{\text{l-o}}$	$1.1^{\text{l-r}}$
157	-	-	-	-	-	-	+	+	+	+	+	+	$1.8^{\text{f-i}}$	$2.4^{\text{d-g}}$
553	-	-	-	-	-	-	-	+	+	+	+	+	$0.9^{\text{m-o}}$	$1.5^{\text{h-o}}$
367	-	-	-	-	-	-	-	+	+	+	+	+	$0.9^{\text{m-o}}$	$2.0^{\text{f-h}}$
18	-	-	-	-	-	-	-	+	+	+	+	+	$1.0^{\text{l-o}}$	$1.6^{\text{h-m}}$
6^{x}	-	-	-	-	-	-	-	-	+	+	+	+		
616	-	-	-	-	-	-	-	-	-	+	+	+	3.1^{b}	3.7^{a}
517	-	-	-	-	-	-	-	-	-	+	+	+	2.8^{bc}	
390	-	-	-	-	-	-	-	-	-	-	-	+	3.5^{a}	$2.9^{\text{b-d}}$
405	-	-	-	-	-	-	-	-	-	-	-	+	$1.9^{\text{e-h}}$	$2.7^{\text{c-e}}$
Horizon	-	-	-	-	-	-	-	-	-	-	-	-	2.8^{bc}	$2.9^{\text{b-d}}$
Fla. 8602	+	+	+	+	+	+	+	+	+	+	+	+	1.7^{gj}	
Tygress	-	/	/	/	/	/	/	/	/	-	-	-		$1.9^{\text{g-k}}$
HA3074	-	/	/	/	/	/	/	/	/	-	-	-	$0.4^{\text{p-s}}$	
Security 28	-	/	/	/	/	/	/	/	/	-	-	-		$1.5^{\text{h-o}}$

[z] + = homozygous *Solanum chilense* , / = heterozygous, - = homozygous *S. lycopersicum*

[Y] Different superscript letters represent statistically significant differences at $P < 0.05$ based on Duncan's multiple range test.

[X] RIL was not tested in Fall 2007 or Spring 2008; evaluation in Fall 2008 indicated susceptibility (data not shown).
