# Supplementary material for: The Tomato Yellow Leaf Curl Virus Resistance Genes Ty-1 and Ty-3 Are Allelic and Code for DFDGD-Class RNA–Dependent RNA Polymerases
Source: PLoS Genet. 2013 Mar 28;9(3):e1003399. doi: 10.1371/journal.pgen.1003399 (PMC3610679; doi:10.1371/journal.pgen.1003399)
Supplement: Table S3 — Tomato plants from the Ty-3 fine-mapping population selected for recombination in the Ty-3 region of chromosome 6, mean Tomato Yellow Leaf Curl Virus (TYLCV) disease severity of their cuttings, and their genotype throughout the region. (PDF) [file pgen.1003399.s009.pdf]

Supplemental table 3. Tomato plants from the *Ty-3* fine-mapping population selected for recombination in the *Ty-3* region of chromosome 6, mean Tomato Yellow Leaf Curl Virus (TYLCV) disease severity of their cuttings, and their genotype thoughout the region.

			Chromosome 6 Introgressed Segment[Z]																																												
Sub-population	Plant	DSI[Y]	**Mi23**	C2At2g39690	SL10401	C2_At4g01900	C2_At5g61510	TG231	T1456	TG97	T0892	C2At3g10920	U231369	T1563	**T0774_UF**	SL_2.40ch06_30.280	SL_2.40ch06_30.696	EcoRI0039C03	HBa0161K22	FOS0169A13	SL_2.40ch06_30.891	HBa0045I03	HBa0135O04	EcoRI0020O03	cLEG-31-P16	FOS0262B11.2	HBa0142H09	SL_2.40ch06_31.040	MboI0074O20	FOS0245D20	SL_2.40ch06_31.097	MboI46E04.1	MboI142A14	FOS9D21	HBa117N23	TG590 F3R3	Spr2	cLET-1-I13)	**P6-25**	SL_2.40ch06_31.660	C2_At3g11210	SL_2.40ch06_31.770	T0507	SL_2.40ch06_31.928	T1079	TG118L	T1098	**T0834**
A	E944-22	1.1	/	/	/	/	/	/	/	/	/	/	/	/	/	/	+	+	+	+	+	+	+	+	+	+	+	+	+	+	+	+	+	+	+	+	+	+	+	+	+	+	+	+	-	-	-	-
A	E944-288	1.3	+	+	+	+	+	+	+	+	+	+	+	+	+	+	+	+	+	+	+	/	/	/	/	/	/	/	/	/	/	/	/	/	/	/	/	/	/	/	/	/	/	/	-	-	-	-
A	E944-355	2.2	+	+	+	+	+	+	+	+	+	+	+	+	/	/	/	/	/	/	/	/	/	/	/	/	/	/	/	/	/	/	/	/	/	/	/	/	/	/	/	/	/	/	-	-	-	-
A	E944-399	2.3	+	+	+	+	/	/	/	/	/	/	/	/	/	/	/	/	/	/	/	/	/	/	/	/	/	/	/	/	/	/	/	/	/	/	/	/	/	/	/	/	/	/	-	-	-	-
A	E944-510	1.6	/	/	+	+	+	+	+	+	+	+	+	+	+	+	+	+	+	+	+	+	+	+	+	+	+	+	+	+	+	+	+	+	+	+	+	+	+	+	+	+	+	+	-	-	-	-
A	E944-604	1.4	+	+	+	+	+	+	+	+	+	+	+	+	+	+	+	+	+	+	+	+	+	+	+	+	+	+	+	+	+	+	+	+	+	/	/	/	/	/	/	/	/	/	-	-	-	-
A	E945-20	1.9	-	-	/	/	/	/	/	/	/	/	/	/	/	/	/	/	/	/	/	/	/	/	/	/	/	/	/	/	/	/	/	/	/	/	/	/	/	/	/	/	/	/	-	-	-	-
A	E945-25	2.8	-	-	-	-	-	-	-	-	/	/	/	/	/	/	/	/	/	/	/	/	/	/	/	/	/	/	/	/	/	/	/	/	/	/	/	/	/	/	/	/	/	/	-	-	-	-
A	E945-102	2.4	+	+	+	+	+	+	+	+	+	+	+	+	/	/	/	/	/	/	/	/	/	/	/	/	/	/	/	/	/	/	/	/	/	/	/	/	/	/	/	/	/	/	-	-	-	-
A	E945-221	1.9	+	+	+	+	+	+	+	+	+	+	+	+	/	/	/	/	/	/	/	/	/	/	/	/	/	/	/	/	/	/	/	/	/	/	/	/	/	/	/	/	/	/	-	-	-	-
A	E945-247	1.3	+	+	+	+	+	+	+	+	+	+	+	+	+	+	+	+	+	+	+	+	+	/	/	/	/	/	/	/	/	/	/	/	/	/	/	/	/	/	/	/	/	/	-	-	-	-
A	E945-254	2.1	-	-	-	-	-	-	-	-	/	/	/	/	/	/	/	/	/	/	/	/	/	/	/	/	/	/	/	/	/	/	/	/	/	/	/	/	/	/	/	/	/	/	-	-	-	-
A	E945-355	1.8	+	+	+	+	+	+	+	+	/	/	/	/	/	/	/	/	/	/	/	/	/	/	/	/	/	/	/	/	/	/	/	/	/	/	/	/	/	/	/	/	/	/	-	-	-	-
A	E945-368	1.5	+	+	+	+	+	+	+	+	+	+	+	+	+	+	+	+	+	+	+	/	/	/	/	/	/	/	/	/	/	/	/	/	/	/	/	/	/	/	/	/	/	/	-	-	-	-
A	E945-374	2.0	+	+	/	/	/	/	/	/	/	/	/	/	/	/	/	/	/	/	/	/	/	/	/	/	/	/	/	/	/	/	/	/	/	/	/	/	/	/	/	/	/	/	-	-	-	-
A	E945-554	2.4	-	-	-	-	-	-	-	-	-	-	-	-	/	/	/	/	/	/	/	/	/	/	/	/	/	/	/	/	/	/	/	/	/	/	/	/	/	/	/	/	/	/	-	-	-	-
A	E945-597	2.3	+	+	+	+	+	+	+	+	/	/	/	/	/	/	/	/	/	/	/	/	/	/	/	/	/	/	/	/	/	/	/	/	/	/	/	/	/	/	/	/	/	/	-	-	-	-
A	E945-612	2.4	-	-	-	-	-	-	-	-	-	-	-	-	-	.	/	/	/	/	/	/	/	/	/	/	/	/	/	/	/	/	/	/	/	/	/	/	/	/	/	/	/	/	-	-	-	-
A	E945-644	3.9	/	/	/	/	/	/	/	/	-	-	-	-	-	-	-	-	-	-	-	-	-	-	-	-	-	-	-	-	-	-	-	-	-	-	-	-	-	-	-	-	-	-	-	-	-	-
A	E945-651	2.3	-	-	/	/	/	/	/	/	/	/	/	/	/	/	/	/	/	/	/	/	/	/	/	/	/	/	/	/	/	/	/	/	/	/	/	/	/	/	/	/	/	/	-	-	-	-
A	E946-153	1.8	+	+	+	+	+	+	+	+	+	+	+	+	/	/	/	/	/	/	/	/	/	/	/	/	/	/	/	/	/	/	/	/	/	/	/	/	/	/	/	/	/	/	-	-	-	-
A	E946-163	1.0	/	/	/	/	/	/	/	/	+	+	+	+	+	+	+	+	+	+	+	+	+	+	+	+	+	+	+	+	+	+	+	+	+	+	+	+	+	+	+	+	+	+	-	-	-	-
A	E946-171	1.1	/	/	+	+	+	+	+	+	+	+	+	+	+	+	+	+	+	+	+	+	+	+	+	+	+	+	+	+	+	+	+	+	+	+	+	+	+	+	+	+	+	+	-	-	-	-
A	E946-250	4.0	/	/	-	-	-	-	-	-	-	-	-	-	-	-	-	-	-	-	-	-	-	-	-	-	-	-	-	-	-	-	-	-	-	-	-	-	-	-	-	-	-	-	-	-	-	-
A	E946-251	2.4	-	-	-	-	-	-	-	-	-	-	-	-	/	/	/	/	/	/	/	/	/	/	/	/	/	/	/	/	/	/	/	/	/	/	/	/	/	/	/	/	/	/	-	-	-	-
A	E946-285	2.4	+	+	/	/	/	/	/	/	/	/	/	/	/	/	/	/	/	/	/	/	/	/	/	/	/	/	/	/	/	/	/	/	/	/	/	/	/	/	/	/	/	/	-	-	-	-
A	E946-304	2.1	-	-	/	/	/	/	/	/	/	/	/	/	/	/	/	/	/	/	/	/	/	/	/	/	/	/	/	/	/	/	/	/	/	/	/	/	/	/	/	/	/	/	-	-	-	-
A	E946-451	1.6	+	+	+	+	+	+	+	+	+	+	+	+	+	/	/	/	/	/	/	/	/	/	/	/	/	/	/	/	/	/	/	/	/	/	/	/	/	/	/	/	/	/	-	-	-	-
A	E946-485	1.8	+	+	/	/	/	/	/	/	/	/	/	/	/	/	/	/	/	/	/	/	/	/	/	/	/	/	/	/	/	/	/	/	/	/	/	/	/	/	/	/	/	/	-	-	-	-
A	E946-511	1.1	/	/	/	/	/	/	/	/	+	+	+	+	+	+	+	+	+	+	+	+	+	+	+	+	+	+	+	+	+	+	+	+	+	+	+	+	+	+	+	+	+	+	-	-	-	-
A	E947-14	1.2	+	+	+	+	+	+	+	+	+	+	+	+	+	/	/	/	/	/	/	/	/	/	/	/	/	/	/	/	/	/	/	/	/	/	/	/	/	/	/	/	/	/	-	-	-	-
A	E947-53	1.8	-	-	/	/	/	/	/	/	/	/	/	/	/	/	/	/	/	/	/	/	/	/	/	/	/	/	/	/	/	/	/	/	/	/	/	/	/	/	/	/	/	/	-	-	-	-
A	E947-317	1.0	/	/	+	+	+	+	+	+	+	+	+	+	+	+	+	+	+	+	+	+	+	+	+	+	+	+	+	+	+	+	+	+	+	+	+	+	+	+	+	+	+	+	-	-	-	-
A	E947-356	4.0	/	/	-	-	-	-	-	-	-	-	-	-	-	-	-	-	-	-	-	-	-	-	-	-	-	-	-	-	-	-	-	-	-	-	-	-	-	-	-	-	-	-	-	-	-	-
A	E947-370	1.6	-	-	-	-	-	-	-	-	-	-	-	/	/	/	/	/	/	/	/	/	/	/	/	/	/	/	/	/	/	/	/	/	/	/	/	/	/	/	/	/	/	/	-	-	-	-
A	E947-383	1.8	+	+	+	+	+	+	+	+	/	/	/	/	/	/	/	/	/	/	/	/	/	/	/	/	/	/	/	/	/	/	/	/	/	/	/	/	/	/	/	/	/	/	-	-	-	-
A	E947-387	4.0	/	/	-	-	-	-	-	-	-	-	-	-	-	-	-	-	-	-	-	-	-	-	-	-	-	-	-	-	-	-	-	-	-	-	-	-	-	-	-	-	-	-	-	-	-	-
A	E947-558	4.0	/	/	-	-	-	-	-	-	-	-	-	-	-	-	-	-	-	-	-	-	-	-	-	-	-	-	-	-	-	-	-	-	-	-	-	-	-	-	-	-	-	-	-	-	-	-
A	E947-588	3.8	-	-	-	-	-	-	-	-	-	-	-	-	-	-	-	-	-	-	-	-	-	-	-	/	/	/	/	/	/	/	/	/	/	/	/	/	/	/	/	/	/	/	-	-	-	-
A	E947-589	1.8	/	/	/	/	/	/	/	/	/	/	/	/	/	/	/	/	/	/	/	/	/	/	/	/	/	/	/	+	+	+	+	+	+	+	+	+	+	+	+	+	+	+	-	-	-	-
A	E947-602	1.8	-	-	-	-	-	-	-	-	/	/	/	/	/	/	/	/	/	/	/	/	/	/	/	/	/	/	/	/	/	/	/	/	/	/	/	/	/	/	/	/	/	/	-	-	-	-
A	E947-617	1.3	/	/	+	+	+	+	+	+	+	+	+	+	+	+	+	+	+	+	+	+	+	+	+	+	+	+	+	+	+	+	+	+	+	+	+	+	+	+	+	+	+	+	-	-	-	-
A	E947-625	1.0	+	+	+	+	+	+	+	+	+	+	+	+	+	+	/	/	/	/	/	/	/	/	/	/	/	/	/	/	/	/	/	/	/	/	/	/	/	/	/	/	/	/	-	-	-	-
A	E947-633	1.0	+	+	/	/	/	/	/	/	/	/	/	/	/	/	/	/	/	/	/	/	/	/	/	/	/	/	/	/	/	/	/	/	/	/	/	/	/	/	/	/	/	/	-	-	-	-
A	E947-713	1.6	/	/	/	/	/	/	/	/	/	/	/	-	-	-	-	-	-	-	-	-	-	-	-	-	-	-	-	-	-	-	-	-	-	-	-	-	-	-	-	-	-	-	-	-	-	-
A	E947-735	1.4	-	-	-	-	-	-	-	-	-	-	-	-	-	.	/	/	/	/	/	/	/	/	/	/	/	/	/	/	/	/	/	/	/	/	/	/	/	/	/	/	/	/	-	-	-	-
A	E947-757	1.0	+	+	/	/	/	/	/	/	/	/	/	/	/	/	/	/	/	/	/	/	/	/	/	/	/	/	/	/	/	/	/	/	/	/	/	/	/	/	/	/	/	/	-	-	-	-
A	E947-809	3.5	/	/	/	/	/	/	/	/	-	-	-	-	-	-	-	-	-	-	-	-	-	-	-	-	-	-	-	-	-	-	-	-	-	-	-	-	-	-	-	-	-	-	-	-	-	-
B	E940-37	2.5	+	+	+	+	+	+	+	+	+	+	+	+	+	/	/	/	/	/	/	/	/	/	/	/	/	/	/	/	/	/	/	/	/	/	/	/	/	/	/	/	/	/	-	-	-	-
B	E940-61	1.8	+	+	/	/	/	/	/	/	/	/	/	/	/	/	/	/	/	/	/	/	/	/	/	/	/	/	/	/	/	/	/	/	/	/	/	/	/	/	/	/	/	/	-	-	-	-
B	E940-81	1.9	-	-	/	/	/	/	/	/	/	/	/	/	/	/	/	/	/	/	/	/	/	/	/	/	/	/	/	/	/	/	/	/	/	/	/	/	/	/	/	/	/	/	-	-	-	-
B	E940-238	3.0	+	+	+	+	+	+	+	+	.	/	/	/	/	/	/	/	/	/	/	/	/	/	/	/	/	/	/	/	/	/	/	/	/	/	/	/	/	/	/	/	/	/	-	-	-	-
B	E940-254	2.5	+	+	/	/	/	/	/	/	/	/	/	/	/	/	/	/	/	/	/	/	/	/	/	/	/	/	/	/	/	/	/	/	/	/	/	/	/	/	/	/	/	/	-	-	-	-
B	E940-302	2.4	-	-	-	-	-	-	-	-	-	-	-	-	-	.	/	/	/	/	/	/	/	/	/	/	/	/	/	/	/	/	/	/	/	/	/	/	/	/	/	/	/	/	-	-	-	-
B	E940-326	1.3	/	/	+	+	+	+	+	+	+	+	+	+	+	+	+	+	+	+	+	+	+	+	+	+	+	+	+	+	+	+	+	+	+	+	+	+	+	+	+	+	+	+	-	-	-	-
B	E940-397	2.1	+	+	+	+	+	+	+	+	+	+	+	/	/	/	/	/	/	/	/	/	/	/	/	/	/	/	/	/	/	/	/	/	/	/	/	/	/	/	/	/	/	/	-	-	-	-
B	E940-406	3.6	-	-	-	-	-	-	-	-	.	/	/	/	/	/	/	/	/	/	/	/	/	/	/	/	/	/	/	/	/	/	/	/	/	/	/	/	/	/	/	/	/	/	-	-	-	-
B	E940-523	3.8	-	-	-	-	-	-	-	-	-	-	-	-	-	-	-	-	-	-	-	-	-	-	-	-	-	-	-	-	-	-	-	-	-	/	/	/	/	/	/	/	/	/	-	-	-	-
B	E940-528	1.8	+	+	+	+	+	+	+	+	.	/	/	/	/	/	/	/	/	/	/	/	/	/	/	/	/	/	/	/	/	/	/	/	/	/	/	/	/	/	/	/	/	/	-	-	-	-
B	E940-541	3.8	-	-	-	-	-	-	-	-	-	-	-	-	-	-	-	-	-	-	-	-	-	-	-	-	-	-	-	-	-	-	-	-	-	-	-	-	/	/	/	/	/	/	-	-	-	-
B	E940-561	2.4	+	+	/	/	/	/	/	/	/	/	/	/	/	/	/	/	/	/	/	/	/	/	/	/	/	/	/	/	/	/	/	/	/	/	/	/	/	/	/	/	/	/	-	-	-	-
B	E940-765	2.4	/	/	/	/	/	/	/	/	/	/	/	/	/	/	+	+	+	+	+	+	+	+	+	+	+	+	+	+	+	+	+	+	+	+	+	+	+	+	+	+	+	+	-	-	-	-
B	E940-770	2.4	+	+	/	/	/	/	/	/	/	/	/	/	/	/	/	/	/	/	/	/	/	/	/	/	/	/	/	/	/	/	/	/	/	/	/	/	/	/	/	/	/	/	-	-	-	-
B	E940-777	2.3	/	/	+	+	+	+	+	+	+	+	+	+	+	+	+	+	+	+	+	+	+	+	+	+	+	+	+	+	+	+	+	+	+	+	+	+	+	+	+	+	+	+	-	-	-	-
B	E940-800	2.0	/	/	+	+	+	+	+	+	+	+	+	+	+	+	+	+	+	+	+	+	+	+	+	+	+	+	+	+	+	+	+	+	+	+	+	+	+	+	+	+	+	+	-	-	-	-
B	E940-837	2.3	+	+	+	+	+	+	+	+	+	+	+	+	+	+	+	/	/	/	/	/	/	/	/	/	/	/	/	/	/	/	/	/	/	/	/	/	/	/	/	/	/	/	-	-	-	-
B	E940-855	2.2	/	/	/	/	/	/	/	/	/	/	/	.	/	/	/	/	/	/	/	/	/	/	/	/	-	-	-	-	-	-	-	-	-	-	-	-	-	-	-	-	-	-	-	-	-	-
B	E940-881	2.0	-	-	-	-	-	-	-	-	-	-	-	-	-	-	/	/	/	/	/	/	/	/	/	/	/	/	/	/	/	/	/	/	/	/	/	/	/	/	/	/	/	/	-	-	-	-
B	E940-918	2.3	+	+	+	+	+	+	+	+	+	+	+	+	+	+	+	+	+	+	/	/	/	/	/	/	/	/	/	/	/	/	/	/	/	/	/	/	/	/	/	/	/	/	-	-	-	-
B	E940-1068	2.1	+	+	/	/	/	/	/	/	/	/	/	/	/	/	/	/	/	/	/	/	/	/	/	/	/	/	/	/	/	/	/	/	/	/	/	/	/	/	/	/	/	/	-	-	-	-

Sub-population	Plant	DSI[Y]	Mi23	C2At2g39690	SL10401	C2_At4g01900	C2_At5g61510	TG231	T1456	TG97	T0892	C2At3g10920	U231369	T1563	T0774_UF	SL_2.40ch06_30.28(	SL_2.40ch06_30.69(	EcoRI0039C03	HBa0161K22	FOS00169A13	SL_2.40ch06_30.891	HBa0045I03	HBa0135O04	EcoRI0020O03	cLEG-31-P16	FOS0262B11.2	HBa0142H09	SL_2.40ch06_31.04(	MboI0074O20	FOS0245D20	SL_2.40ch06_31.097	MboI46E04.1	MboI142A14	FOS9D21	HBa117N23	TG590 F3R3	Spr2	cLET-1-I13)	P6-25	SL_2.40ch06_31.66(	C2_At3g11210	SL_2.40ch06_31.77(	T0507	SL_2.40ch06_31.928	T1079	TG118L	T1098	T0834
B	E941-33	2.5	/	/	/	/	/	/	/	/	.	.	+	+	+	+	+	+	+	+	+	+	+	+	+	+	+	+	+	+	+	+	+	+	+	+	+	+	+	+	+	+	+	+	-	-	-	-
B	E941-42	2.8	/	/	+	+	+	+	+	+	+	+	+	+	+	+	+	+	+	+	+	+	+	+	+	+	+	+	+	+	+	+	+	+	+	+	+	+	+	+	+	+	+	+	-	-	-	-
B	E941-110	3.1	+	+	+	+	+	+	+	+	+	+	+	+	+	.	/	/	/	/	/	/	/	/	/	/	/	/	/	/	/	/	/	/	/	/	/	/	/	/	/	/	/	/	-	-	-	-
B	E941-164	2.8	-	-	-	-	-	-	-	-	-	-	-	-	-	-	-	/	/	/	/	/	/	/	/	/	/	/	/	/	/	/	/	/	/	/	/	/	/	/	/	/	/	/	-	-	-	-
B	E941-174	3.3	/	/	-	-	-	-	-	-	-	-	-	-	-	-	-	-	-	-	-	-	-	-	-	-	-	-	-	-	-	-	-	-	-	-	-	-	-	-	-	-	-	-	-	-	-	-
B	E941-175	1.6	/	/	/	/	/	/	/	/	/	/	/	/	+	+	+	+	+	+	+	+	+	+	+	+	+	+	+	+	+	+	+	+	+	+	+	+	+	+	+	+	+	+	-	-	-	-
B	E941-194	2.0	-	-	/	/	/	/	/	/	/	/	/	/	/	/	/	/	/	/	/	/	/	/	/	/	/	/	/	/	/	/	/	/	/	/	/	/	/	/	/	/	/	/	-	-	-	-
B	E941-209	2.5	+	+	+	+	+	+	+	+	+	+	+	+	/	/	/	/	/	/	/	/	/	/	/	/	/	/	/	/	/	/	/	/	/	/	/	/	/	/	/	/	/	/	-	-	-	-
B	E941-249	2.1	-	-	/	/	/	/	/	/	/	/	/	/	/	/	/	/	/	/	/	/	/	/	/	/	/	/	/	/	/	/	/	/	/	/	/	/	/	/	/	/	/	/	-	-	-	-
B	E941-255	2.1	/	/	/	/	/	/	/	/	.	.	+	+	+	+	+	+	+	+	+	+	+	+	+	+	+	+	+	+	+	+	+	+	+	+	+	+	+	+	+	+	+	+	-	-	-	-
B	E941-285	3.5	-	-	-	-	-	-	-	-	-	-	-	-	-	-	.	/	/	/	/	/	/	/	/	/	/	/	/	/	/	/	/	/	/	/	/	/	/	/	/	/	/	/	-	-	-	-
B	E941-310	4.0	/	/	/	/	/	/	/	/	/	/	/	/	/	/	-	-	-	-	-	-	-	-	-	-	-	-	-	-	-	-	-	-	-	-	-	-	-	-	-	-	-	-	-	-	-	-
B	E941-383	3.4	+	+	+	+	+	+	+	+	+	+	+	+	+	.	/	/	/	/	/	/	/	/	/	/	/	/	/	/	/	/	/	/	/	/	/	/	/	/	/	/	/	/	-	-	-	-
B	E941-398	2.9	/	/	/	/	/	/	/	/	/	/	/	.	+	+	+	+	+	+	+	+	+	+	+	+	+	+	+	+	+	+	+	+	+	+	+	+	+	+	+	+	+	+	-	-	-	-
B	E941-450	4.0	-	-	-	-	-	-	-	-	-	-	-	-	-	-	-	-	-	-	-	-	-	-	-	-	-	-	-	-	-	-	-	-	-	-	/	/	/	/	/	/	/	/	-	-	-	-
B	E941-459	3.5	+	.	/	/	/	/	/	/	/	/	/	/	/	/	/	/	/	/	/	/	/	/	/	/	/	/	/	/	/	/	/	/	/	/	/	/	/	/	/	/	/	/	-	-	-	-
B	E941-515	2.9	-	-	/	/	/	/	/	/	/	/	/	/	/	/	/	/	/	/	/	/	/	/	/	/	/	/	/	/	/	/	/	/	/	/	/	/	/	/	/	/	/	/	-	-	-	-
B	E941-555	3.3	/	/	/	/	/	/	/	/	.	+	+	+	+	+	+	+	+	+	+	+	+	+	+	+	+	+	+	+	+	+	+	+	+	+	+	+	+	+	+	+	+	+	-	-	-	-
B	E941-558	3.5	/	/	/	/	/	/	/	/	/	/	/	/	/	/	/	/	/	/	/	/	/	/	+	+	+	+	+	+	+	+	+	+	+	+	+	+	+	+	+	+	+	+	-	-	-	-
B	E941-573	2.4	/	/	/	/	/	/	/	/	/	/	/	/	+	+	+	+	+	+	+	+	+	+	+	+	+	+	+	+	+	+	+	+	+	+	+	+	+	+	+	+	+	+	-	-	-	-
B	E941-602	3.5	-	-	-	-	-	-	-	-	-	-	-	-	-	-	/	/	/	/	/	/	/	/	/	/	/	/	/	/	/	/	/	/	/	/	/	/	/	/	/	/	/	/	-	-	-	-
B	E941-812	2.1	+	.	/	/	/	/	/	/	/	/	/	/	/	/	/	/	/	/	/	/	/	/	/	/	/	/	/	/	/	/	/	/	/	/	/	/	/	/	/	/	/	/	-	-	-	-
B	E941-816	2.1	/	/	/	/	/	/	/	/	/	/	/	/	+	+	+	+	+	+	+	+	+	+	+	+	+	+	+	+	+	+	+	+	+	+	+	+	+	+	+	+	+	+	-	-	-	-
B	E941-899	2.3	+	.	/	/	/	/	/	/	/	/	/	/	/	/	/	/	/	/	/	/	/	/	/	/	/	/	/	/	/	/	/	/	/	/	/	/	/	/	/	/	/	/	-	-	-	-
B	E942-10	2.4	+	+	+	+	+	+	+	+	+	+	+	+	+	/	/	/	/	/	/	/	/	/	/	/	/	/	/	/	/	/	/	/	/	/	/	/	/	/	/	/	/	/	-	-	-	-
B	E942-19	1.0	/	/	+	+	+	+	+	+	+	+	+	+	+	+	+	+	+	+	+	+	+	+	+	+	+	+	+	+	+	+	+	+	+	+	+	+	+	+	+	+	+	+	-	-	-	-
B	E942-20	2.8	/	/	/	/	/	/	/	/	/	/	/	/	/	/	+	+	+	+	+	+	+	+	+	+	+	+	+	+	+	+	+	+	+	+	+	+	+	+	+	+	+	+	-	-	-	-
B	E942-103	3.0	/	/	/	/	/	/	/	/	.	+	+	+	+	+	+	+	+	+	+	+	+	+	+	+	+	+	+	+	+	+	+	+	+	+	+	+	+	+	+	+	+	+	-	-	-	-
B	E942-127	3.9	-	-	/	/	/	/	/	/	/	/	/	/	/	/	/	/	/	/	/	/	/	/	/	/	/	/	/	/	/	/	/	/	/	/	/	/	/	/	/	/	/	/	-	-	-	-
B	E942-162	2.4	/	/	/	/	/	/	/	/	/	/	/	/	/	/	/	/	/	/	/	/	/	/	/	/	/	/	/	/	/	/	/	/	/	/	/	/	+	+	+	+	+	+	-	-	-	-
B	E942-165	2.3	/	/	/	.	.	+	+	+	+	+	+	+	+	+	+	+	+	+	+	+	+	+	+	+	+	+	+	+	+	+	+	+	+	+	+	+	+	+	+	+	+	+	-	-	-	-
B	E942-202	2.9	-	-	/	/	/	/	/	/	/	/	/	/	/	/	/	/	/	/	/	/	/	/	/	/	/	/	/	/	/	/	/	/	/	/	/	/	/	/	/	/	/	/	-	-	-	-
B	E942-242	3.0	+	+	+	+	/	/	/	/	/	/	/	/	/	/	/	/	/	/	/	/	/	/	/	/	/	/	/	/	/	/	/	/	/	/	/	/	/	/	/	/	/	/	-	-	-	-
B	E942-396	3.0	+	+	+	+	+	+	+	+	+	+	+	+	+	/	/	/	/	/	/	/	/	/	/	/	/	/	/	/	/	/	/	/	/	/	/	/	/	/	/	/	/	/	-	-	-	-
B	E942-402	2.9	-	-	-	-	-	-	-	-	-	-	-	-	/	/	/	/	/	/	/	/	/	/	/	/	/	/	/	/	/	/	/	/	/	/	/	/	/	/	/	/	/	/	-	-	-	-
B	E942-411	2.8	/	/	/	/	/	/	/	/	/	/	/	/	/	/	/	/	/	/	/	/	/	/	/	/	/	/	/	/	/	/	/	/	/	/	+	+	+	+	+	+	+	+	-	-	-	-
B	E942-425	2.8	/	/	/	/	/	/	/	/	/	/	/	/	/	/	/	/	/	/	/	/	/	/	/	/	/	/	/	/	/	/	/	/	/	/	.	-	-	-	-	-	-	-	-	-	-	-
B	E942-482	2.3	+	+	+	+	+	+	+	+	+	+	+	+	+	+	+	+	+	+	/	/	/	/	/	/	/	/	/	/	/	/	/	/	/	/	/	/	/	/	/	/	/	/	-	-	-	-
B	E942-601	3.1	-	-	-	-	-	-	-	-	-	-	-	-	-	-	-	/	/	/	/	/	/	/	/	/	/	/	/	/	/	/	/	/	/	/	/	/	/	/	/	/	/	/	-	-	-	-
B	E942-603	2.8	-	-	-	-	-	-	-	-	.	/	/	/	/	/	/	/	/	/	/	/	/	/	/	/	/	/	/	/	/	/	/	/	/	/	/	/	/	/	/	/	/	/	-	-	-	-
B	E942-665	4.0	/	/	-	-	-	-	-	-	-	-	-	-	-	-	-	-	-	-	-	-	-	-	-	-	-	-	-	-	-	-	-	-	-	-	-	-	-	-	-	-	-	-	-	-	-	-
B	E942-717	3.1	+	+	/	/	/	/	/	/	/	/	/	/	/	/	/	/	/	/	/	/	/	/	/	/	/	/	/	/	/	/	/	/	/	/	/	/	/	/	/	/	/	/	-	-	-	-
B	E942-719	3.9	-	-	-	-	-	-	-	-	-	-	-	-	-	-	-	-	-	-	-	-	-	-	-	-	-	-	-	-	-	-	-	-	-	-	-	/	/	/	/	/	/	/	-	-	-	-
B	E942-773	2.5	/	/	/	/	+	+	+	+	+	+	+	+	+	+	+	+	+	+	+	+	+	+	+	+	+	+	+	+	+	+	+	+	+	+	+	+	+	+	+	+	+	+	-	-	-	-
B	E942-979	1.1	/	/	+	+	+	+	+	+	+	+	+	+	+	+	+	+	+	+	+	+	+	+	+	+	+	+	+	+	+	+	+	+	+	+	+	+	+	+	+	+	+	+	-	-	-	-
B	E942-1017	1.6	+	+	+	+	+	+	+	+	+	+	+	+	/	/	/	/	/	/	/	/	/	/	/	/	/	/	/	/	/	/	/	/	/	/	/	/	/	/	/	/	/	/	-	-	-	-
B	E942-1019	2.0	-	-	-	/	/	/	/	/	/	/	/	/	/	/	/	/	/	/	/	/	/	/	/	/	/	/	/	/	/	/	/	/	/	/	/	/	/	/	/	/	/	/	-	-	-	-
B	E942-1078	1.6	/	/	+	+	+	+	+	+	+	+	+	+	+	+	+	+	+	+	+	+	+	+	+	+	+	+	+	+	+	+	+	+	+	+	+	+	+	+	+	+	+	+	-	-	-	-
B	E942-1204	2.3	/	/	/	/	/	/	/	/	/	/	/	/	/	/	/	+	+	+	+	+	+	+	+	+	+	+	+	+	+	+	+	+	+	+	+	+	+	+	+	+	+	+	-	-	-	-
B	E942-1205	1.5	/	/	+	+	+	+	+	+	+	+	+	+	+	+	+	+	+	+	+	+	+	+	+	+	+	+	+	+	+	+	+	+	+	+	+	+	+	+	+	+	+	+	-	-	-	-
B	E943-51	2.9	-	-	-	-	-	-	-	-	-	-	-	/	/	/	/	/	/	/	/	/	/	/	/	/	/	/	/	/	/	/	/	/	/	/	/	/	/	/	/	/	/	/	-	-	-	-
B	E943-111	3.2	/	/	/	/	/	/	/	/	/	/	/	/	/	/	+	+	+	+	+	+	+	+	+	+	+	+	+	+	+	+	+	+	+	+	+	+	+	+	+	+	+	+	-	-	-	-
B	E943-118	1.5	/	/	/	/	/	/	/	/	+	+	+	+	+	+	+	+	+	+	+	+	+	+	+	+	+	+	+	+	+	+	+	+	+	+	+	+	+	+	+	+	+	+	-	-	-	-
B	E943-140	2.0	/	/	/	/	+	+	+	+	+	+	+	+	+	+	+	+	+	+	+	+	+	+	+	+	+	+	+	+	+	+	+	+	+	+	+	+	+	+	+	+	+	+	-	-	-	-
B	E943-141	2.8	/	/	/	/	/	/	/	/	/	/	/	/	+	+	+	+	+	+	+	+	+	+	+	+	+	+	+	+	+	+	+	+	+	+	+	+	+	+	+	+	+	+	-	-	-	-
B	E943-156	3.8	-	-	-	-	-	-	-	-	/	/	/	/	/	/	/	/	/	/	/	/	/	/	/	/	/	/	/	/	/	/	/	/	/	/	/	/	/	/	/	/	/	/	-	-	-	-
B	E943-165	3.0	+	+	+	+	+	+	+	+	+	+	+	+	+	+	/	/	/	/	/	/	/	/	/	/	/	/	/	/	/	/	/	/	/	/	/	/	/	/	/	/	/	/	-	-	-	-
B	E943-428	3.5	-	-	-	-	-	-	-	-	/	/	/	/	/	/	/	/	/	/	/	/	/	/	/	/	/	/	/	/	/	/	/	/	/	/	/	/	/	/	/	/	/	/	-	-	-	-
B	E943-452	2.3	/	/	+	+	+	+	+	+	+	+	+	+	+	+	+	+	+	+	+	+	+	+	+	+	+	+	+	+	+	+	+	+	+	+	+	+	+	+	+	+	+	+	-	-	-	-
B	E943-488	1.7	-	-	-	-	-	-	-	-	-	-	-	-	/	/	/	/	/	/	/	/	/	/	/	/	/	/	/	/	/	/	/	/	/	/	/	/	/	/	/	/	/	/	-	-	-	-
B	E943-505	2.0	-	-	-	-	/	/	/	/	/	/	/	/	/	/	/	/	/	/	/	/	/	/	/	/	/	/	/	/	/	/	/	/	/	/	/	/	/	/	/	/	/	/	-	-	-	-
B	E943-524	1.8	/	/	/	/	/	/	/	/	+	+	+	+	+	+	+	+	+	+	+	+	+	+	+	+	+	+	+	+	+	+	+	+	+	+	+	+	+	+	+	+	+	+	-	-	-	-
B	E943-611	2.5	/	/	+	+	+	+	+	+	+	+	+	+	+	+	+	+	+	+	+	+	+	+	+	+	+	+	+	+	+	+	+	+	+	+	+	+	+	+	+	+	+	+	-	-	-	-
B	E943-621	2.4	+	+	+	+	/	/	/	/	/	/	/	/	/	/	/	/	/	/	/	/	/	/	/	/	/	/	/	/	/	/	/	/	/	/	/	/	/	/	/	/	/	/	-	-	-	-
B	E943-851	2.4	+	+	+	+	+	+	+	+	+	+	+	+	+	+	+	+	+	+	+	+	+	+	+	+	+	+	+	+	+	+	+	+	+	+	+	+	/	/	/	/	/	/	-	-	-	-
B	E943-962	3.8	/	/	/	/	/	/	/	/	/	/	/	-	-	-	-	-	-	-	-	-	-	-	-	-	-	-	-	-	-	-	-	-	-	-	-	-	-	-	-	-	-	-	-	-	-	-
B	E943-994	3.3	+	+	+	+	+	+	+	+	/	/	/	/	/	/	/	/	/	/	/	/	/	/	/	/	/	/	/	/	/	/	/	/	/	/	/	/	/	/	/	/	/	/	-	-	-	-
B	E943-1027	2.1	+	+	/	/	/	/	/	/	/	/	/	/	/	/	/	/	/	/	/	/	/	/	/	/	/	/	/	/	/	/	/	/	/	/	/	/	/	/	/	/	/	/	-	-	-	-
B	E943-1030	1.5	/	/	+	+	+	+	+	+	+	+	+	+	+	+	+	+	+	+	+	+	+	+	+	+	+	+	+	+	+	+	+	+	+	+	+	+	+	+	+	+	+	+	-	-	-	-
B	E943-1032	2.6	/	/	/	/	/	/	/	/	/	/	/	/	/	/	/	/	/	/	/	/	/	/	/	/	/	/	/	/	/	/	/	/	/	/	/	-	-	-	-	-	-	-	-	-	-	-

Sub-population	Plant	DSI[Y]	Mi23	C2At2g39690	SL10401	C2_At4g01900	C2_At5g61510	TG231	T1456	TG97	T0892	C2At3g10920	U231369	T1563	T0774_UF	SL_2.40ch06_30.280	SL_2.40ch06_30.696	EcoRI0039C03	HBa0161K22	FOS00169A13	SL_2.40ch06_30.891	HBa0045I03	HBa0135O04	EcoRI0020O03	cLEG-31-P16	FOS0262B11.2	HBa0142H09	SL_2.40ch06_31.040	MboI0074O20	FOS0245D20	SL_2.40ch06_31.097	MboI46E04.1	MboI142A14	FOS9D21	HBa117N23	TG590 F3R3	Spr2	cLET-1-I13)	P6-25	SL_2.40ch06_31.660	C2_At3g11210	SL_2.40ch06_31.770	T0507	SL_2.40ch06_31.928	T1079	TG118L	T1098	T0834
B	E943-1043	4.0	-	-	-	-	-	-	-	-	-	-	-	-	-	-	-	-	-	-	-	-	-	-	-	-	-	-	-	-	-	-	-	-	-	-	-	-	/	/	/	/	/	/	-	-	-	-
B	E943-1291	2.8	+	+	+	+	+	+	+	+	+	+	+	+	/	/	/	/	/	/	/	/	/	/	/	/	/	/	/	/	/	/	/	/	/	/	/	/	/	/	/	/	/	/	-	-	-	-
B	E943-1302	1.1	/	/	/	/	/	/	/	/	/	/	/	/	+	+	+	+	+	+	+	+	+	+	+	+	+	+	+	+	+	+	+	+	+	+	+	+	+	+	+	+	+	+	-	-	-	-
C	E948-14	1.8	-	-	-	-	-	-	-	-	-	-	-	-	+	+	+	+	+	+	+	+	+	+	+	+	+	+	+	+	+	+	+	+	+	+	+	+	+	+	+	+	+	+	+	+	/	/
C	E948-39	2.9	-	-	-	-	-	-	-	-	-	-	-	-	/	/	/	/	/	/	/	/	/	/	/	/	/	/	/	/	/	/	/	/	/	/	/	/	/	/	/	/	/	/	/	/	/	+
C	E948-50	2.2	-	-	-	-	-	-	-	-	-	-	-	-	/	/	/	/	/	/	-	-	-	-	-	-	-	-	-	-	-	-	-	-	-	-	-	-	-	-	-	-	-	-	-	-	-	-
C	E948-120	1.6	-	-	-	-	-	-	-	-	-	-	-	-	/	/	/	/	/	/	/	/	/	/	/	/	/	/	/	/	/	/	/	/	/	/	/	/	/	/	/	/	/	/	-	-	-	-
C	E948-168	1.4	-	-	-	-	-	-	-	-	-	-	-	-	+	+	+	+	+	+	+	+	+	+	+	+	+	+	+	+	+	+	+	+	+	+	+	+	+	+	+	+	+	+	+	+	+	/
C	E948-251	2.3	-	-	-	-	-	-	-	-	-	-	-	-	/	/	/	/	/	/	/	/	/	/	/	/	/	/	/	/	/	/	/	/	/	/	/	/	/	/	/	/	/	/	/	/	/	-
C	E948-280	3.1	-	-	-	-	-	-	-	-	-	-	-	-	/	/	/	/	/	/	/	/	/	/	/	/	/	/	/	/	/	/	/	/	/	/	/	/	/	/	/	/	/	/	-	-	-	-
C	E948-281	2.0	-	-	-	-	-	-	-	-	-	-	-	-	/	/	/	+	+	+	+	+	+	+	+	+	+	+	+	+	+	+	+	+	+	+	+	+	+	+	+	+	+	+	+	+	+	+
C	E948-292	1.9	-	-	-	-	-	-	-	-	-	-	-	-	+	+	+	+	+	+	+	+	+	+	+	+	+	+	+	+	+	+	+	+	+	+	+	+	+	+	+	+	+	+	+	.	/	/
C	E948-321	2.5	-	-	-	-	-	-	-	-	-	-	-	-	/	/	/	/	/	/	/	/	/	/	/	/	/	/	/	/	/	/	/	/	/	/	/	/	/	/	/	/	/	/	/	/	/	+
C	E948-341	2.9	-	-	-	-	-	-	-	-	-	-	-	-	/	/	/	/	/	/	/	/	/	/	/	/	/	/	/	/	/	/	/	/	/	/	/	/	/	/	/	/	/	/	/	/	/	+
C	E948-351	2.0	-	-	-	-	-	-	-	-	-	-	-	-	/	/	/	/	/	/	/	/	/	/	/	/	/	/	/	/	/	/	/	/	/	/	/	/	/	/	/	/	/	/	/	/	-	-
C	E948-375	1.8	-	-	-	-	-	-	-	-	-	-	-	-	/	+	+	+	+	+	+	+	+	+	+	+	+	+	+	+	+	+	+	+	+	+	+	+	+	+	+	+	+	+	+	+	+	+
C	E948-647	2.3	-	-	-	-	-	-	-	-	-	-	-	-	/	/	/	/	/	/	/	/	/	/	/	/	/	/	/	/	/	/	/	/	/	/	/	/	/	/	/	/	/	/	/	/	/	-
C	E948-693	2.1	-	-	-	-	-	-	-	-	-	-	-	-	/	/	/	/	/	/	/	/	/	/	/	/	/	/	/	/	/	/	/	/	/	/	/	/	/	/	/	/	/	/	/	/	/	+
C	E948-697	2.3	-	-	-	-	-	-	-	-	-	-	-	-	/	/	/	/	/	/	/	/	/	/	/	/	/	/	/	/	/	/	/	/	/	/	/	+	+	+	+	+	+	+	+	+
C	E948-721	2.5	-	-	-	-	-	-	-	-	-	-	-	-	/	/	/	/	/	/	/	/	/	/	/	/	/	/	/	/	/	/	/	-	-	-	-	-	-	-	-	-	-	-	-	-
C	E948-725	3.0	-	-	-	-	-	-	-	-	-	-	-	-	-	-	-	-	-	/	/	/	/	/	/	/	/	/	/	/	/	/	/	/	/	/	/	/	/	/	/	/	/	/	/	/	/	/
C	E948-739	2.3	-	-	-	-	-	-	-	-	-	-	-	-	/	/	/	/	/	/	/	/	/	/	/	/	/	/	/	/	/	/	/	/	/	/	/	/	/	/	/	/	/	/	/	/	/	+
C	E948-770	2.0	-	-	-	-	-	-	-	-	-	-	-	-	+	+	+	+	+	+	+	+	+	+	+	+	+	+	+	+	+	+	+	+	+	+	+	+	+	+	+	+	+	+	+	/	/	/
C	E948-811	1.8	-	-	-	-	-	-	-	-	-	-	-	-	/	/	/	/	/	/	/	/	/	/	/	/	/	/	/	/	/	/	/	/	/	/	/	/	/	/	/	/	/	/	-	-	-	-
C	E948-874	1.8	-	-	-	-	-	-	-	-	-	-	-	-	/	/	/	/	/	/	/	/	/	/	/	/	/	/	/	-	-	-	-	-	-	-	-	-	-	-	-	-	-	-	-	-	-	-	-
C	E948-882	1.4	-	-	-	-	-	-	-	-	-	-	-	-	+	+	+	+	+	+	+	+	+	+	+	+	+	+	+	+	+	+	+	+	+	+	+	+	+	+	+	+	+	+	+	+	/	/
C	E948-944	2.1	-	-	-	-	-	-	-	-	-	-	-	-	+	+	+	+	+	+	+	+	+	+	+	+	+	+	+	+	+	+	+	+	+	+	+	+	/	/	/	/	/	/	/	/
C	E948-950	1.0	-	-	-	-	-	-	-	-	-	-	-	-	+	+	+	+	+	+	+	+	+	+	+	+	+	+	+	+	+	+	+	+	+	+	+	+	/	/	/	/	/	/	/	/
C	E948-1002	2.8	-	-	-	-	-	-	-	-	-	-	-	-	/	/	/	/	/	/	/	/	/	/	/	/	/	/	/	/	/	/	/	/	/	/	/	/	/	/	/	/	/	+	+	+
C	E948-1057	1.5	-	-	-	-	-	-	-	-	-	-	-	-	/	/	/	/	/	/	/	/	/	/	/	/	/	/	/	/	/	/	/	/	/	/	/	/	/	/	/	/	+	+	+	+
C	E948-1066	1.6	-	-	-	-	-	-	-	-	-	-	-	-	+	+	+	+	+	+	+	+	+	+	+	+	+	+	+	+	+	+	+	+	+	+	+	+	+	+	+	+	+	+	+	+	/	/
C	E948-1076	1.6	-	-	-	-	-	-	-	-	-	-	-	-	/	/	/	/	/	/	/	/	/	/	/	/	/	/	/	/	/	/	/	/	/	/	/	/	/	/	/	/	/	-	-	-
C	E948-1146	2.8	-	-	-	-	-	-	-	-	-	-	-	-	+	+	+	/	/	/	/	/	/	/	/	/	/	/	/	/	/	/	/	/	/	/	/	/	/	/	/	/	/	/	/	/	/	-
C	E948-1243	2.5	-	-	-	-	-	-	-	-	-	-	-	-	/	/	/	/	/	/	+	+	+	+	+	+	+	+	+	+	+	+	+	+	+	+	+	+	+	+	+	+	+	+	+	+	+	+
C	E949-95	2.1	-	-	-	-	-	-	-	-	-	-	-	-	+	+	+	+	+	+	+	+	+	+	+	+	+	+	+	+	+	+	+	+	+	+	+	+	+	+	+	+	+	+	+	+	+	/
C	E949-148	2.1	-	-	-	-	-	-	-	-	-	-	-	-	+	+	+	+	+	+	+	+	+	+	+	+	+	+	+	+	+	+	+	+	+	+	+	+	+	+	+	+	+	+	+	+	/	/
C	E949-403	4.0	-	-	-	-	-	-	-	-	-	-	-	-	-	-	-	-	-	-	-	-	-	-	-	-	-	-	-	-	-	-	-	-	-	-	-	-	-	-	-	-	-	-	-	-	-	/
C	E949-481	2.4	-	-	-	-	-	-	-	-	-	-	-	-	+	+	+	+	+	+	+	+	+	+	+	+	+	+	+	+	+	+	+	+	+	+	+	+	+	+	+	+	+	+	+	+	+	/
C	E949-678	2.4	-	-	-	-	-	-	-	-	-	-	-	-	+	+	+	+	+	+	+	+	+	+	+	+	+	+	+	+	+	+	+	+	+	+	+	+	+	+	+	+	+	+	/	/	/	/
C	E949-686	2.8	-	-	-	-	-	-	-	-	-	-	-	-	.	.	/	/	/	/	/	/	/	/	/	/	/	/	/	/	/	/	/	/	/	/	/	/	/	/	/	/	/	/	/	/	/	/
C	E949-782	3.0	-	-	-	-	-	-	-	-	-	-	-	-	/	/	/	/	/	/	/	/	/	/	/	/	/	/	/	/	/	/	/	/	/	/	/	/	/	/	/	/	/	/	/	/	/	-
C	E949-787	4.0	-	-	-	-	-	-	-	-	-	-	-	-	-	-	-	-	-	-	-	-	-	-	-	-	-	-	-	-	-	-	-	-	-	-	-	-	-	-	-	-	/	/	/	/	/	/
C	E949-806	2.8	-	-	-	-	-	-	-	-	-	-	-	-	+	+	+	/	/	/	/	/	/	/	/	/	/	/	/	/	/	/	/	/	/	/	/	/	/	/	/	/	/	/	/	/	/	/
C	E949-885	2.2	-	-	-	-	-	-	-	-	-	-	-	-	+	+	+	+	+	+	+	+	+	+	+	+	/	/	/	/	/	/	/	/	/	/	/	/	/	/	/	/	/	/	/	/	/	/
C	E949-1023	2.6	-	-	-	-	-	-	-	-	-	-	-	-	/	/	/	/	/	/	/	/	/	/	/	/	/	/	/	/	/	/	/	/	/	/	+	+	+	+	+	+	+	+	+	+	+	+
C	E949-1026	3.0	-	-	-	-	-	-	-	-	-	-	-	-	/	/	/	/	/	/	/	/	+	+	+	+	+	+	+	+	+	+	+	+	+	+	+	+	+	+	+	+	+	+	+	+	+	+	+	+
C	E949-1266	2.1	-	-	-	-	-	-	-	-	-	-	-	-	/	/	/	/	/	/	/	/	/	/	/	/	/	/	/	/	/	/	/	/	/	/	/	/	/	/	/	/	/	/	/	/	/	-
C	E949-1283	2.8	-	-	-	-	-	-	-	-	-	-	-	-	-	.	/	/	/	/	/	/	/	/	/	/	/	/	/	/	/	/	/	/	/	/	/	/	/	/	/	/	/	/	/	/	/	/
C	E949-1284	4.0	-	-	-	-	-	-	-	-	-	-	-	-	-	-	-	-	-	-	-	-	-	-	-	-	-	-	-	-	-	-	-	-	-	-	-	/	/	/	/	/	/	/	/	/
C	E949-1332	2.0	-	-	-	-	-	-	-	-	-	-	-	-	/	/	/	/	/	/	/	/	/	/	/	/	-	-	-	-	-	-	-	-	-	-	-	-	-	-	-	-	-	-	-	-	-	-
C	E949-1338	1.6	-	-	-	-	-	-	-	-	-	-	-	-	+	+	+	+	+	+	+	+	+	+	+	+	+	+	+	+	+	+	+	+	+	+	+	+	+	+	+	+	+	+	/	/	/	/
C	E949-1351	2.4	-	-	-	-	-	-	-	-	-	-	-	-	/	/	/	/	/	/	/	/	/	/	/	/	/	/	/	/	/	/	/	/	/	/	/	/	/	/	/	/	/	/	-	-	-	-
C	E949-1393	4.0	-	-	-	-	-	-	-	-	-	-	-	-	-	-	-	-	-	-	-	-	-	-	-	-	-	-	-	-	-	-	-	-	-	-	-	-	-	-	-	-	-	-	/	/	/	/
C	E950-100	4.0	-	-	-	-	-	-	-	-	-	-	-	-	-	-	-	-	-	-	-	-	-	-	-	-	-	-	-	-	-	-	-	-	-	-	-	-	-	-	-	-	-	-	-	-	-	/
C	E950-107	2.4	-	-	-	-	-	-	-	-	-	-	-	-	/	/	/	/	/	/	/	/	/	/	/	/	/	/	/	/	/	/	/	/	/	/	/	/	/	/	/	/	/	/	/	/	/	/
C	E950-141	1.5	-	-	-	-	-	-	-	-	-	-	-	-	+	+	+	+	+	+	+	+	+	+	+	+	+	+	+	+	+	+	+	+	+	+	+	+	+	+	+	+	+	+	/	/	/	/
C	E950-165	4.0	-	-	-	-	-	-	-	-	-	-	-	-	-	-	-	-	-	-	-	-	-	-	-		/	/	/	/	/	/	/	/	/	/	/	/	/	/	/	/	/	/	/	/	/	/
C	E950-220	3.3	-	-	-	-	-	-	-	-	-	-	-	-	/	/	/	/	/	/	/	/	/	/	/	/	/	/	/	/	/	/	/	/	/	/	/	/	/	/	/	/	/	/	/	/	/	+
C	E950-293	1.8	-	-	-	-	-	-	-	-	-	-	-	-	+	+	+	+	+	+	+	+	+	+	+	+	+	+	+	+	+	+	+	+	+	+	+	+	+	+	+	+	+	+	+	+	+	/
C	E950-365	4.0	-	-	-	-	-	-	-	-	-	-	-	-	-	-	-	-	-	-	-	-	-	-	-	-	-	-	-	-	-	-	-	-	-	/	/	/	/	/	/	/	/	/	/	/	/	/
C	E950-375	2.0	-	-	-	-	-	-	-	-	-	-	-	-	/	/	/	/	/	/	/	/	/	/	/	/	/	/	/	/	/	/	/	/	/	/	+	+	+	+	+	+	+	+	+	+	+	+
C	E950-426	2.3	-	-	-	-	-	-	-	-	-	-	-	-	+	+	+	+	+	+	+	+	+	+	+	+	+	+	+	+	+	+	+	+	+	/	/	/	/	/	/	/	/	/	/	/	/	/
C	E950-437	1.6	-	-	-	-	-	-	-	-	-	-	-	-	+	+	+	+	+	+	+	+	+	+	+	+	+	+	+	+	+	+	+	+	+	+	+	+	+	+	+	+	+	/	/	/	/	/
C	E950-449	2.6	-	-	-	-	-	-	-	-	-	-	-	-	+	+	+	+	+	+	+	+	+	+	+	+	+	+	+	+	+	+	+	+	+	+	+	+	+	+	+	+	+	+	/	/	/	/
C	E950-533	2.1	-	-	-	-	-	-	-	-	-	-	-	-	+	+	+	+	+	+	+	+	+	+	+	+	+	+	+	+	+	+	+	+	+	+	+	+	+	+	+	+	+	+	+	+	+	/
C	E950-551	2.0	-	-	-	-	-	-	-	-	-	-	-	-	/	/	/	/	/	/	/	/	/	/	/	/	/	/	/	/	/	/	/	/	/	/	/	+	+	+	+	+	+	+	+	+
C	E950-628	2.9	-	-	-	-	-	-	-	-	-	-	-	-	-	.	/	/	/	/	/	/	/	/	/	/	/	/	/	/	/	/	/	/	/	/	/	/	/	/	/	/	/	/	/	/	/	/
C	E950-648	2.4	-	-	-	-	-	-	-	-	-	-	-	-	/	/	/	/	/	/	/	/	/	/	/	/	/	/	/	/	/	/	/	/	/	/	/	/	/	/	/	/	/	/	/	/	/	-
C	E950-715	4.0	-	-	-	-	-	-	-	-	-	-	-	-	/	/	/	-	-	-	-	-	-	-	-	-	-	-	-	-	-	-	-	-	-	-	-	-	-	-	-	-	-	-	-	-	-	-
C	E950-732	2.0	-	-	-	-	-	-	-	-	-	-	-	-	+	+	+	+	+	+	+	+	+	+	+	+	+	+	+	+	+	+	+	+	+	+	+	/	/	/	/	/	/	/	/	/

Sub-population	Plant	DSI[Y]	**Mi23**	C2At2g39690	SL10401	C2_At4g01900	C2_At5g61510	TG231	T1456	TG97	T0892	C2At3g10920	U231369	T1563	**T0774_UF**	SL_2.40ch06_30.280	SL_2.40ch06_30.696	EcoRI0039C03	HBa0161K22	FOS00169A13	SL_2.40ch06_30.891	HBa0045I03	HBa0135O04	EcoRI0020O03	cLEG-31-P16	FOS0262B11.2	HBa0142H09	SL_2.40ch06_31.040	MboI0074O20	FOS0245D20	SL_2.40ch06_31.097	MboI46E04.1	MboI142A14	FOS9D21	HBa117N23	TG590 F3R3	Spr2	cLET-1-I13)	**P6-25**	SL_2.40ch06_31.660	C2_At3g11210	SL_2.40ch06_31.770	T0507	SL_2.40ch06_31.928	T1079	TG118L	T1098	**T0834**
C	E950-733	2.3	-	-	-	-	-	-	-	-	-	-	-	-	/	/	/	/	/	/	/	/	/	/	/	/	/	/	/	/	/	/	/	/	/	/	/	/	/	/	/	/	/	/	/	+	+	+
C	E950-811	4.0	-	-	-	-	-	-	-	-	-	-	-	-	-	-	-	-	-	-	-	-	-	-	-	-	-	-	-	-	-	-	-	-	-	-	-	-	-	-	-	-	-	-	/	/	/	/
C	E950-827	2.1	-	-	-	-	-	-	-	-	-	-	-	-	/	/	/	/	/	/	/	/	/	/	/	/	/	/	/	/	/	/	/	/	/	/	/	/	/	/	/	/	/	/	/	/	/	+
C	E950-930	1.8	-	-	-	-	-	-	-	-	-	-	-	-	+	+	+	+	+	+	+	+	+	+	+	+	+	+	+	+	+	+	+	+	+	+	+	+	+	+	+	+	+	+	/	/	/	/
C	E950-952	2.8	-	-	-	-	-	-	-	-	-	-	-	-	+	/	/	/	/	/	/	/	/	/	/	/	/	/	/	/	/	/	/	/	/	/	/	/	/	/	/	/	/	/	/	/	/	/
C	E950-1000	2.3	-	-	-	-	-	-	-	-	-	-	-	-	/	/	/	/	/	/	/	/	/	/	/	/	/	/	/	/	/	/	/	/	/	/	/	/	/	/	/	/	/	/	/	-	-	-
C	E950-1010	4.0	-	-	-	-	-	-	-	-	-	-	-	-	-	-	-	-	-	-	-	-	-	-	-	-	-	-	-	/	/	/	/	/	/	/	/	/	/	/	/	/	/	/	/	/	/	/
C	E950-1027	2.3	-	-	-	-	-	-	-	-	-	-	-	-	+	+	+	+	+	+	+	+	+	+	+	+	+	+	+	+	+	+	+	+	+	+	+	+	+	+	+	+	+	+	/	/	/	/
C	E950-1029	3.8	-	-	-	-	-	-	-	-	-	-	-	-	-	-	-	-	-	-	-	-	-	-	-	-	-	-	-	-	-	-	-	-	-	-	-	-	-	-	-	-	-	-	-	-	/	/
C	E950-1031	2.6	-	-	-	-	-	-	-	-	-	-	-	-	/	/	/	/	/	/	/	/	/	/	/	/	/	/	/	/	/	/	/	/	/	/	/	/	/	/	/	/	/	/	+	+	+	+
C	E950-1035	4.0	-	-	-	-	-	-	-	-	-	-	-	-	-	-	-	-	-	-	-	-	-	-	-	-	-	-	-	-	-	-	-	-	-	-	-	-	-	-	-	-	-	-	-	-	/	/
C	E950-1049	4.0	-	-	-	-	-	-	-	-	-	-	-	-	-	-	-	-	-	-	-	-	-	-	-	-	-	-	-	-	-	-	-	-	-	-	/	/	/	/	/	/	/	/	/	/	/	/
C	E950-1079	1.6	-	-	-	-	-	-	-	-	-	-	-	-	+	+	+	+	+	+	+	+	+	+	+	+	+	+	+	+	+	+	+	+	+	+	+	+	+	+	+	+	+	+	/	/	/	/
C	E950-1122	4.0	-	-	-	-	-	-	-	-	-	-	-	-	-	-	-	-	-	-	-	-	-	-	-	-	-	-	-	/	/	/	/	/	/	/	/	/	/	/	/	/	/	/	/	/	/	/
C	E950-1153	1.7	-	-	-	-	-	-	-	-	-	-	-	-	+	+	+	+	+	+	+	+	+	+	+	+	+	+	+	+	+	+	+	+	+	+	/	/	/	/	/	/	/	/	/	/	/	/
C	E950-1245	4.0	-	-	-	-	-	-	-	-	-	-	-	-	-	-	-	-	-	-	-	-	-	-	-	-	-	-	-	-	-	-	-	-	-	-	-	-	-	-	-	-	-	-	-	-	-	/
C	E950-1270	4.0	-	-	-	-	-	-	-	-	-	-	-	-	-	-	-	-	-	-	-	-	-	-	-	-	-	-	-	-	-	-	-	-	-	-	-	-	-	-	-	-	-	-	-	-	-	/
C	E950-1286	3.0	-	-	-	-	-	-	-	-	-	-	-	-	-	-	-	/	/	/	/	/	/	/	/	/	/	/	/	/	/	/	/	/	/	/	/	/	/	/	/	/	/	/	/	/	/	/
C	E951-44	2.5	-	-	-	-	-	-	-	-	-	-	-	-	/	/	/	/	/	/	/	/	/	/	/	/	/	/	/	/	/	/	/	/	/	/	/	/	/	/	/	/	/	/	+	+	+	+
C	E951-105	1.9	-	-	-	-	-	-	-	-	-	-	-	-	+	+	+	+	+	+	+	+	+	+	+	+	+	+	+	+	+	+	+	+	+	+	+	+	+	+	+	+	+	+	+	+	+	/
C	E951-107	3.9	-	-	-	-	-	-	-	-	-	-	-	-	-	-	-	-	-	-	-	-	-	-	-	-	-	-	-	-	-	-	-	-	-	-	-	-	-	-	-	-	-	-	.	/	/	/
C	E951-127	1.0	-	-	-	-	-	-	-	-	-	-	-	-	+	+	+	+	+	+	+	+	+	+	+	+	+	+	+	+	+	+	+	+	+	+	+	+	+	+	+	+	+	+	+	/	/	/
C	E951-133	2.0	-	-	-	-	-	-	-	-	-	-	-	-	/	/	/	/	/	/	/	/	/	/	/	/	/	/	/	/	/	/	/	/	/	/	/	/	/	-	-	-	-	-	-	-	-	-
C	E951-165	3.1	-	-	-	-	-	-	-	-	-	-	-	-	/	/	/	/	/	/	/	/	/	/	/	/	/	/	/	/	/	/	/	/	/	/	/	/	/	-	-	-	-	-	-	-	-	-
C	E951-230	4.0	-	-	-	-	-	-	-	-	-	-	-	-	/	.	-	-	-	-	-	-	-	-	-	-	-	-	-	-	-	-	-	-	-	-	-	-	-	-	-	-	-	-	-	-	-	-
C	E951-287	3.4	-	-	-	-	-	-	-	-	-	-	-	-	/	/	/	/	/	/	/	/	/	/	/	/	/	/	/	/	/	/	/	/	/	/	/	/	/	+	+	+	+	+	+	+	+	+
C	E951-303	2.0	-	-	-	-	-	-	-	-	-	-	-	-	/	.	+	+	+	+	+	+	+	+	+	+	+	+	+	+	+	+	+	+	+	+	+	+	+	+	+	+	+	+	+	+	+	+
C	E951-339	4.0	-	-	-	-	-	-	-	-	-	-	-	-	-	-	-	-	-	-	-	-	-	-	-	-	-	-	-	-	-	-	-	-	-	-	-	-	-	-	-	-	-	-	-	-	-	/
C	E951-361	1.8	-	-	-	-	-	-	-	-	-	-	-	-	+	+	+	+	+	+	+	+	+	+	+	+	+	+	+	+	+	+	+	+	+	+	+	+	+	+	+	+	+	+	+	+	+	/
C	E951-370	2.0	-	-	-	-	-	-	-	-	-	-	-	-	/	/	/	/	/	/	/	/	/	/	/	/	/	/	/	/	/	/	/	/	/	/	/	/	/	/	/	/	/	/	/	/	/	-
C	E951-403	2.7	-	-	-	-	-	-	-	-	-	-	-	-	/	/	/	/	/	/	/	/	/	/	+	+	+	+	+	+	+	+	+	+	+	+	+	+	+	+	+	+	+	+	+	+	+	+
C	E951-428	2.7	-	-	-	-	-	-	-	-	-	-	-	-	/	/	/	/	/	/	/	/	/	/	/	/	/	/	/	-	-	-	-	-	-	-	-	-	-	-	-	-	-	-	-	-	-	-
C	E951-429	2.9	-	-	-	-	-	-	-	-	-	-	-	-	/	/	/	/	/	/	/	/	/	/	/	/	/	/	/	/	/	/	/	/	/	/	/	/	/	/	/	/	/	-	-	-	-	-
C	E951-431	2.6	-	-	-	-	-	-	-	-	-	-	-	-	/	/	/	/	/	/	/	/	/	/	/	/	/	/	/	/	/	/	/	/	/	/	/	/	/	/	/	/	/	/	/	+	+	+
C	E951-453	1.6	-	-	-	-	-	-	-	-	-	-	-	-	/	/	/	+	+	+	+	+	+	+	+	+	+	+	+	+	+	+	+	+	+	+	+	+	+	+	+	+	+	+	+	+	+	+
C	E951-485	4.0	-	-	-	-	-	-	-	-	-	-	-	-	-	-	-	-	-	-	-	-	-	-	-	-	-	-	-	-	-	-	-	-	-	-	-	-	-	-	-	-	-	-	/	/	/	/
C	E951-486	4.0	-	-	-	-	-	-	-	-	-	-	-	-	-	-	-	-	-	-	-	-	-	-	-	-	-	-	-	-	-	-	-	-	-	-	/	/	/	/	/	/	/	/	/	/	/	/
C	E951-554	2.5	-	-	-	-	-	-	-	-	-	-	-	-	/	/	/	/	/	/	/	/	/	/	/	/	/	/	/	/	/	/	/	/	/	/	/	/	/	/	/	/	/	/	/	/	/	+
C	E951-570	2.1	-	-	-	-	-	-	-	-	-	-	-	-	/	.	+	+	+	+	+	+	+	+	+	+	+	+	+	+	+	+	+	+	+	+	+	+	+	+	+	+	+	+	+	+	+	+
C	E951-653	1.1	-	-	-	-	-	-	-	-	-	-	-	-	+	+	+	+	+	+	+	+	+	+	+	+	+	+	+	+	+	+	+	+	+	+	+	+	/	/	/	/	/	/	/	/	/	/
C	E951-776	1.8	-	-	-	-	-	-	-	-	-	-	-	-	+	+	+	+	+	+	+	+	+	+	+	+	+	+	+	+	+	+	+	+	+	+	+	+	+	+	+	+	+	+	/	/	/	/
C	E951-816	1.8	-	-	-	-	-	-	-	-	-	-	-	-	/	.	+	+	+	+	+	+	+	+	+	+	+	+	+	+	+	+	+	+	+	+	+	+	+	+	+	+	+	+	+	+	+	+
C	E951-839	3.1	-	-	-	-	-	-	-	-	-	-	-	-	+	.	/	/	/	/	/	/	/	/	/	/	/	/	/	/	/	/	/	/	/	/	/	/	/	/	/	/	/	/	/	/	/	/
C	E951-860	4.0	-	-	-	-	-	-	-	-	-	-	-	-	/		-	-	-	-	-	-	-	-	-	-	-	-	-	-	-	-	-	-	-	-	-	-	-	-	-	-	-	-	-	-	-	-
C	E951-875	2.8	-	-	-	-	-	-	-	-	-	-	-	-	/	/	/	/	/	/	/	/	/	/	/	/	/	/	/	/	/	/	/	/	/	/	/	/	/	.	.	.	.	.	.	.	+	+
C	E951-878	2.5	-	-	-	-	-	-	-	-	-	-	-	-	/	/	/	/	/	/	/	/	/	/	/	/	/	/	/	/	/	/	/	/	/	/	/	/	/	/	/	/	/	.	.	.	+	+
C	E951-905	2.8	-	-	-	-	-	-	-	-	-	-	-	-	/	/	/	/	/	/	/	/	/	/	/	+	+	+	+	+	+	+	+	+	+	+	+	+	+	+	+	+	+	+	+	+	+	+
C	E951-942	2.4	-	-	-	-	-	-	-	-	-	-	-	-	/	/	/	/	/	/	/	/	/	/	/	/	/	/	/	/	/	/	/	/	/	/	/	/	/	/	+	+	+	+	+	+	+	+
C	E951-952	1.9	-	-	-	-	-	-	-	-	-	-	-	-	/	/	+	+	+	+	+	+	+	+	+	+	+	+	+	+	+	+	+	+	+	+	+	+	+	+	+	+	+	+	+	+	+	+
C	E951-992	2.9	-	-	-	-	-	-	-	-	-	-	-	-	/	/	/	/	/	/	/	/	/	/	/	/	/	/	/	/	/	/	/	/	/	/	/	/	/	/	/	/	/	/	/	-	-	-
C	E951-1018	2.6	-	-	-	-	-	-	-	-	-	-	-	-	+	+	+	+	+	+	+	+	+	+	+	+	+	+	+	+	+	+	+	+	+	/	/	/	/	/	/	/	/	/	/	/	/	/
C	E951-1034	.	-	-	-	-	-	-	-	-	-	-	-	-	-	-	-	-	-	-	-	-	-	-	-	-	-	-	-	-	-	-	-	-	-	-	-	-	-	/	/	/	/	/	/	/	/	/
C	E951-1043	2.6	-	-	-	-	-	-	-	-	-	-	-	-	/	/	/	/	/	/	/	/	/	/	/	/	/	/	/	/	/	/	/	/	/	/	/	/	/	/	/	/	/	/	/	/	/	-
C	E951-1092	4.0	-	-	-	-	-	-	-	-	-	-	-	-	-	-	-	-	-	-	-	-	-	-	-	-	-	-	-	-	-	-	-	-	-	-	-	-	-	-	-	-	-	-	-	-	-	/
C	E951-1112	2.4	-	-	-	-	-	-	-	-	-	-	-	-	+	+	+	+	+	+	+	+	/	/	/	/	/	/	/	/	/	/	/	/	/	/	/	/	/	/	/	/	/	/	/	/	/	/
C	E951-1126	2.9	-	-	-	-	-	-	-	-	-	-	-	-	/	/	/	/	/	/	/	/	/	+	+	+	+	+	+	+	+	+	+	+	+	+	+	+	+	+	+	+	+	+	+	+	+	+
C	E951-1127	4.0	-	-	-	-	-	-	-	-	-	-	-	-	/	.	-	-	-	-	-	-	-	-	-	-	-	-	-	-	-	-	-	-	-	-	-	-	-	-	-	-	-	-	-	-	-	-
C	E951-1178	3.8	-	-	-	-	-	-	-	-	-	-	-	-	-	-	-	-	-	-	-	-	-	-	-	-	-	-	-	-	-	-	-	-	-	-	-	-	-	-	-	-	-	-	-	-	-	/
C	E982-35	2.6	-	-	-	-	-	-	-	-	-	-	-	-	/	/	/	/	/	/	/	/	/	/	/	/	/	/	/	/	/	/	/	/	/	/	/	/	/	/	/	/	/	/	+	+	+	+
C	E982-121	2.3	-	-	-	-	-	-	-	-	-	-	-	-	+	+	+	+	+	+	+	+	+	+	+	+	+	+	+	+	+	+	+	+	+	+	+	+	+	+	+	+	+	+	/	/	/	/
C	E982-128	2.8	-	-	-	-	-	-	-	-	-	-	-	-	-	.	/	/	/	/	/	/	/	/	/	/	/	/	/	/	/	/	/	/	/	/	/	/	/	/	/	/	/	/	/	/	/	/
C	E982-218	2.8	-	-	-	-	-	-	-	-	-	-	-	-	/	/	/	/	/	/	/	/	/	/	/	/	/	/	/	/	/	/	/	/	/	/	/	/	/	/	/	/	/	/	/	/	+	+
C	E982-259	2.5	-	-	-	-	-	-	-	-	-	-	-	-	+	+	+	+	+	+	+	/	/	/	/	/	/	/	/	/	/	/	/	/	/	/	/	/	/	/	/	/	/	/	/	/	/	/
C	E982-276	3.0	-	-	-	-	-	-	-	-	-	-	-	-	/	/	/	/	/	/	/	/	/	/	/	/	/	/	/	/	/	/	/	/	/	/	/	/	/	/	/	/	/	/	/	+	+	+
C	E982-280	4.0	-	-	-	-	-	-	-	-	-	-	-	-	/	.	-	-	-	-	-	-	-	-	-	-	-	-	-	-	-	-	-	-	-	-	-	-	-	-	-	-	-	-	-	-	-	-
C	E982-367	2.9	-	-	-	-	-	-	-	-	-	-	-	-	/	/	/	/	/	/	/	/	/	/	/	/	/	/	/	/	/	/	/	/	/	/	/	/	/	/	/	/	/	/	/	/	/	+
C	E982-369	1.4	-	-	-	-	-	-	-	-	-	-	-	-	+	+	+	+	+	+	+	+	+	+	+	+	+	+	+	+	+	+	+	+	+	+	+	+	+	+	+	+	+	+	/	/	/	/
C	E982-383	4.0	-	-	-	-	-	-	-	-	-	-	-	-	/	/	/	/	-	-	-	-	-	-	-	-	-	-	-	-	-	-	-	-	-	-	-	-	-	-	-	-	-	-	-	-	-	-
C	E982-384	2.3	-	-	-	-	-	-	-	-	-	-	-	-	/	/	/	/	/	/	/	/	/	/	/	/	/	/	/	/	/	/	/	/	/	/	/	/	/	/	/	/	/	/	+	+	+	+
C	E982-432	1.8	-	-	-	-	-	-	-	-	-	-	-	-	+	+	+	+	+	+	+	+	+	+	+	+	+	+	+	+	+	+	+	+	+	+	+	+	+	+	+	+	+	+	/	/	/	/

Sub-population	Plant	DSI[Y]	Mi23	C2At2g39690	SL10401	C2_At4g01900	C2_At5g61510	TG231	T1456	TG97	T0892	C2At3g10920	U231369	T1563	T0774_UF	SL_2.40ch06_30.280	SL_2.40ch06_30.696	EcoRI0039C03	HBa0161K22	FOS00169A13	SL_2.40ch06_30.891	HBa0045I03	HBa0135O04	EcoRI0020O03	cLEG-31-P16	FOS0262B11.2	HBa0142H09	SL_2.40ch06_31.040	MboI0074O20	FOS0245D20	SL_2.40ch06_31.097	MboI46E04.1	MboI142A14	FOS9D21	HBa117N23	TG590 F3R3	Spr2	cLET-1-I13)	P6-25	SL_2.40ch06_31.660	C2_At3g11210	SL_2.40ch06_31.770	T0507	SL_2.40ch06_31.928	T1079	TG118L	T1098	T0834
C	E982-446	2.8	-	-	-	-	-	-	-	-	-	-	-	-	/	/	/	/	/	/	/	/	/	/	/	/	/	/	/	/	/	/	/	/	/	/	/	/	/	/	/	/	/	/	+	+	+	+
C	E982-468	2.0	-	-	-	-	-	-	-	-	-	-	-	-	+	+	+	+	+	+	+	+	+	+	+	+	+	+	+	+	+	+	+	+	+	+	+	+	+	+	+	+	+	+	/	/	/	/
C	E982-533	4.0	-	-	-	-	-	-	-	-	-	-	-	-	-	-	-	-	-	-	-	-	-	-	-	-	-	-	-	-	-	-	-	-	-	-	-	-	-	-	-	-	-	-	/	/	/	/
C	E982-584	2.5	-	-	-	-	-	-	-	-	-	-	-	-	/	/	/	/	/	/	/	/	/	+	+	+	+	+	+	+	+	+	+	+	+	+	+	+	+	+	+	+	+	+	+	+	+	+
C	E982-626	2.4	-	-	-	-	-	-	-	-	-	-	-	-	/	/	/	/	/	/	/	/	/	/	/	/	/	/	/	/	/	/	/	/	/	/	/	/	/	/	/	/	/	/	/	/	/	+
C	E982-690	4.0	-	-	-	-	-	-	-	-	-	-	-	-	-	-	-	-	-	-	-	-	-	-	-	-	-	-	-	-	-	-	-	-	-	-	-	-	-	-	-	-	-	-	-	-	-	/
C	E982-693	2.6	-	-	-	-	-	-	-	-	-	-	-	-	/	/	/	/	/	/	/	/	/	/	-	-	-	-	-	-	-	-	-	-	-	-	-	-	-	-	-	-	-	-	-	-	-	-
C	E982-699	2.1	-	-	-	-	-	-	-	-	-	-	-	-	/	/	/	/	/	/	/	/	/	/	/	/	/	/	/	/	/	/	/	/	/	/	/	/	/	/	/	+	+	+	+	+	+	+
C	E982-721	2.6	-	-	-	-	-	-	-	-	-	-	-	-	-	.	/	/	/	/	/	/	/	/	/	/	/	/	/	/	/	/	/	/	/	/	/	/	/	/	/	/	/	/	/	/	/	/
C	E982-803	2.8	-	-	-	-	-	-	-	-	-	-	-	-	/	/	/	/	/	/	/	/	/	/	/	/	/	/	/	/	/	/	/	/	/	/	/	/	/	/	/	/	/	/	/	/	/	-
C	E982-816	2.1	-	-	-	-	-	-	-	-	-	-	-	-	+	+	+	+	+	+	+	+	+	+	+	+	+	+	+	+	+	+	+	+	+	+	+	+	+	+	+	+	+	+	+	+	+	/
C	E982-818	4.0	-	-	-	-	-	-	-	-	-	-	-	-	-	-	-	-	-	-	-	-	-	-	-	-	-	-	-	-	-	-	-	-	-	-	-	-	-	-	-	-	-	-	-	/	/	/
C	E982-823	4.0	-	-	-	-	-	-	-	-	-	-	-	-	-	-	-	-	-	-	-	-	-	-	-	-	-	-	-	-	-	-	-	-	-	-	-	-	-	-	-	-	-	-	/	/	/	/
C	E982-892	2.6	-	-	-	-	-	-	-	-	-	-	-	-	+	+	+	+	+	+	+	+	+	+	+	+	+	+	+	+	+	+	+	+	+	+	+	+	+	+	+	+	+	+	+	+	+	/
C	E982-915	2.4	-	-	-	-	-	-	-	-	-	-	-	-	/	/	/	/	/	/	/	/	/	/	/	/	/	/	/	/	/	/	/	/	/	/	/	-	-	-	-	-	-	-	-	-	-	-
C	E982-927	2.1	-	-	-	-	-	-	-	-	-	-	-	-	+	+	+	+	+	+	+	+	+	+	+	+	+	+	+	+	+	+	+	+	+	+	+	+	+	+	+	+	+	+	+	+	/	/
C	E982-966	2.1	-	-	-	-	-	-	-	-	-	-	-	-	/	/	/	/	/	/	/	/	/	/	/	/	/	/	/	/	/	/	/	/	/	/	/	/	/	/	/	/	/	/	.	+	+	+
C	E982-1034	2.5	-	-	-	-	-	-	-	-	-	-	-	-	/	/	+	+	+	+	+	+	+	+	+	+	+	+	+	+	+	+	+	+	+	+	+	+	+	+	+	+	+	+	+	+	+	+
C	E982-1055	3.0	-	-	-	-	-	-	-	-	-	-	-	-	/	/	/	/	/	/	/	/	/	/	/	/	/	/	/	/	/	/	/	/	/	/	/	/	/	/	/	/	/	/	/	/	/	-
C	E982-1069	2.6	-	-	-	-	-	-	-	-	-	-	-	-	+	+	+	+	+	+	+	+	+	+	+	+	+	+	+	+	+	+	+	+	+	+	+	+	+	+	+	+	+	+	+	+	/	/
C	E982-1097	1.5	-	-	-	-	-	-	-	-	-	-	-	-	+	+	+	+	+	+	+	+	+	+	+	+	+	+	+	+	+	+	+	+	+	+	+	+	/	/	/	/	/	/	/	/	/	/
C	E982-1098	3.3	-	-	-	-	-	-	-	-	-	-	-	-	-	.	/	/	/	/	/	/	/	/	/	/	/	/	/	/	/	/	/	/	/	/	/	/	/	/	/	/	/	/	/	/	/	/
C	E982-1110	1.6	-	-	-	-	-	-	-	-	-	-	-	-	+	+	+	+	+	+	+	+	+	+	+	+	+	+	+	+	+	+	+	+	+	+	+	+	+	+	+	+	+	+	/	/	/	/
C	E982-1132	2.8	-	-	-	-	-	-	-	-	-	-	-	-	/	/	/	/	/	/	/	/	/	/	/	/	/	/	/	/	/	/	/	/	/	/	/	/	/	/	/	/	/	/	/	/	-	-
C	E982-1148	1.9	-	-	-	-	-	-	-	-	-	-	-	-	+	+	+	+	+	+	+	+	+	+	+	+	+	+	+	+	+	+	+	+	+	+	+	+	+	+	+	+	+	+	/	/	/	/
C	E982-1170	2.0	-	-	-	-	-	-	-	-	-	-	-	-	+	+	+	+	+	+	+	+	+	+	+	+	+	+	+	+	+	+	+	+	+	+	+	+	+	+	+	+	+	+	+	+	/	/
C	E982-1212	4.0	-	-	-	-	-	-	-	-	-	-	-	-	-	-	-	-	-	-	-	-	-	-	-	-	-	-	-	-	-	-	-	-	-	-	-	-	-	-	-	-	-	-	/	/	/	/
C	E982-1245	2.9	-	-	-	-	-	-	-	-	-	-	-	-	/	/	/	/	/	/	/	/	/	/	/	/	/	/	/	/	/	/	/	/	/	/	/	/	/	/	/	/	/	/	+	+	+	+

[Z] + = homozygous *Solanum chilense* , / = heterozygous, - = homozygous *S. lycopersicum, "." = missing data*

[Y] DSI = mean disease severity index as described in the "Materials and Methods"
