# Supplementary material for: The Tomato Yellow Leaf Curl Virus Resistance Genes Ty-1 and Ty-3 Are Allelic and Code for DFDGD-Class RNA–Dependent RNA Polymerases
Source: PLoS Genet. 2013 Mar 28;9(3):e1003399. doi: 10.1371/journal.pgen.1003399 (PMC3610679; doi:10.1371/journal.pgen.1003399)
Supplement: Table S4 — Average Tomato yellow leaf curl virus disease severity index (DSI) for tomato cuttings evaluated in spring 2009. (PDF) [file pgen.1003399.s010.pdf]

Supplemental table 4. Average *Tomato yellow leaf curl virus* disease severity index (DSI) for tomato cuttings evaluated in Spring 2009.

Line	Description	DSI			
Fla. 7776	susceptible parent	4.0 ^{A}	±	0.0	(n=6)
(Fla. 7776 x RIL 554) F_1	heterozygous control	3.3 ^{B}	±	0.1	(n=5)
(Fla. 7776 x RIL 157) F_1	heterozygous control	3.3 ^{B}	±	0.1	(n=6)
RIL 554	resistant parent	1.3 $^{D\text{-}E}$	±	0.2	(n=6)
RIL 157	resistant parent	1.4 ^{D}	±	0.2	(n=5)
Fla. 8680	resistant donor	1.0 ^{E}	±	0.0	(n=6)
Tygress	commercial hybrid	1.1 $^{D\text{-}E}$	±	0.1	(n=6)
Security28	commercial hybrid	1.8 ^{C}	±	0.1	(n=6)
Fla. 8602	resistant control	1.0 ^{E}	±	0.0	(n=4)
Horizon	susceptible control	4.0 ^{A}	±	0.0	(n=4)

Results are displayes as mean ± SE; n = number of cuttings evaluated for each line; DSI = disease severity index as described in the "Materials and Methods" where higher numbers indicate more disease; different superscript letters represent statistically significant differences at $P < 0.05$ based on Duncan's multiple range test.
