# Supplementary material for: The Tomato Yellow Leaf Curl Virus Resistance Genes Ty-1 and Ty-3 Are Allelic and Code for DFDGD-Class RNA–Dependent RNA Polymerases
Source: PLoS Genet. 2013 Mar 28;9(3):e1003399. doi: 10.1371/journal.pgen.1003399 (PMC3610679; doi:10.1371/journal.pgen.1003399)
Supplement: Table S5 — Molecular markers on chromosome 6 of tomato. (PDF) [file pgen.1003399.s011.pdf]

**Supplemental table 5. Molecular markers on chromosome 6 of tomato.**

| Marker name | Marker type | | Primer sequence (5'-3') | Annealing temperature | Restriction enzyme | Reference | Location on Tomato WGS Chromosomes (SL2.40) | Letter used in figure 2 |
|---|---|---|---|---|---|---|---|---|
| M-H309K01 | CAPS | F | AGCCCCCAGAAAGACTTGTT | 60°C | *Hpy* F3I | Verlaan 2011 | SL2.40ch06:3571844..3571863 | |
| | | R | TTTTTAAGGGGTGTGCCAAG | | | | SL2.40ch06:3571219..3571238 | |
| T1563 | CAPS | F | ACTTCACCTACAAATCCTTCCAGA | 56°C | *Taq* I | Ji 2007 | SL2.40ch06:29260641..29260664 | |
| | | R | GCCCTTCCCAATCCAGCAGT | | | | SL2.40ch06:29259047..29259066 | |
| T0774 | CAPS | F | CTCCTCAACTGGCTAATTCTCAGG | 55°C | *MnI* I | Ji 2007 | SL2.40ch06:30027683..30027706 | |
| | | R | GCAAACCAAATACATCGCGTATCAC | | | | SL2.40ch06:30027028..30027052 | |
| MSc05732-3 | CAPS | F | ATGCTTTTCGAGCACGAGCCT | 55°C | *Rsa* I | Verlaan 2011 | SL2.40ch06:30523528..30523548 | |
| | | R | AGCCTAAAGAGAACTAGGCAGGGGA | | | | SL2.40ch06:30524212..30524236 | |
| MSc05732-4 | CAPS | F | ACGAGATGGAGCGGTCTTCAAGCT | 55°C | *Dde* | Verlaan 2011 | SL2.40ch06:30600857..30600880 | |
| | | R | GACAGATCTCCCGGTAGGAGAGCA | | | | SL2.40ch06:30601501..30601524 | |
| SL_2.40ch06_30.696 (Ty-3-M1) | CAPS | F | TCTGAATGCAGGGTAATCATGT | 60°C | Msp I | Hutton 2011 | SL2.40ch06:30696326..30696347 | |
| | | R | TGATTTCAGGCCTCATAGAACC | | | | SL2.40ch06:30696915..30696936 | |
| HBa0161K22 (Ty-3-M3) | CAPS | F | AGCTCTTCCAGGAGCAGTTTG | 58°C | Mse I | Hutton 2011 | SL2.40ch06:30814757..30814777 | A |
| | | R | CCCTCCAAATAGTTTCACAAATACG | | | | SL2.40ch06:30815261..30815285 | |
| UF_TY3-P1 | CAPS | F | AAGGAGGATCTGGCTGCTTT | 55 °C | Alu I | Present | SL2.40ch06:30816304..30816323 | B |
| | | R | GTTGGTTTGAATCGCCTTCT | | | | SL2.40ch06:30816698..30816717 | |
| UF_TY3-P3 | CAPS | F | TCAATTTTCACTGTTCCTGACT | 55 °C | BstN I | Present | SL2.40ch06:30821240..30821261 | C |
| | | R | CAAGAGTGAGGTTGTTCTTGATG | | | | SL2.40ch06:30821517..30821539 | |
| WU_M17 | Sequence based | F | CCCCCTTAGGAACATTCGTCCTCA | 55°C | n/a | Present | SL2.40ch06:30824901-30826100 | D |
| | | R | AGGGTAGGGAACAAGCCAAGGCA | | | | SL2.40ch06:30826113..30826135 | |
| FOS00169A13 (Ty-3-M4) | CAPS | F | AGCTATCAGCTGCCAGAGACAT | 56°C | Mse I | Hutton 2011 | SL2.40ch06:30852074..30852095 | E |
| | | R | CACCATCATTGTATCCAGAGAGC | | | | SL2.40ch06:30852469..30852492 | |
| WUR_M25 | Sequence based | F | TGCCAGACTCAGCATTAGTTTGGGG | 55°C | n/a | Present | SL2.40ch06:30868214..30868238 | F |
| | | R | TGTCCCCATCATGCCACACTTCCA | | | | SL2.40ch06:30869157..30869180 | |
| UF_TY3-P18 | CAPS | F | AAGGTGCGGAGTGAAATTCT | 55 °C | Hinf I | Present | SL2.40ch06:30872101-30873200 | G |
| | | R | TTGGTGAGGCGTTGATACAC | | | | SL2.40ch06:30873067..30873086 | |
| WU_M27 | Sequence based | F | TGTCATCTCCCAGGGCTCTCTGT | 55°C | n/a | Present | SL2.40ch06:30875560..30875582 | H |
| | | R | ACCTGTGGTGAAGGTAGTGCGGA | | | | SL2.40ch06:30876226..30876248 | |
| UF_TY3-P19 | SCAR | F | CGTTCTGCTTAATGTGGCAAT | 55 °C | n/a | Present | SL2.40ch06:30876061..30876081 | I |
| | | R | CAACGGAGGGAGCATATCAT | | | | SL2.40ch06:30876451..30876470 | |
| WU_M29 | Sequence based | F | TCTACACTATGAGCCACTGCTCGT | 55°C | n/a | Present | SL2.40ch06:30880703..30880726 | J |
| | | R | TCCTGAATCGGCCTCTGATTTGGA | | | | SL2.40ch06:30881414..30881437 | |
| WU_M31 | Sequence based | F | GCCTGGACGAATGGGAGGCAC | 55°C | n/a | Present | SL2.40ch06:30885455..30885475 | K |
| | | R | ATGGGCATCGGTCACTCGCG | | | | SL2.40ch06:30886383..30886402 | |
| UF_TY3-P23 | CAPS | F | CAGAAAGGGCCGATGAATAA | 55 °C | Alu I | Present | SL2.40ch06:30886934..30886953 | L |
| | | R | GCGCAAATGGAGGTAAAAAG | | | | SL2.40ch06:30887313..30887332 | |
| UF_TY3-P24 | CAPS | F | TGGTCCTCCCTTAACCATTG | 55 °C | Dpn II | Present | SL2.40ch06:30890519..30890538 | M |
| | | R | TGGAACCGTGAGGAAGAAAC | | | | SL2.40ch06:30890878..30890897 | |
| SL_2.40ch06_30.891 (Ty-3-M5) | CAPS | F | CCGAAGGTGATAACCACGAC | 56°C | BstN I | Hutton 2011 | SL2.40ch06:30891965..30891984 | |
| | | R | GCGACCCTAACCAACAATAAAC | | | | SL2.40ch06:30891379..30891400 | |
| SL_2.40ch06_31.040 | CAPS | F | TTCCTCCAAGTCATCCACCTAC | 56°C | HpyCH4 IV | Hutton 2011 | SL2.40ch06:3104393..31040415 | |
| | | R | GAGTCGTTTATTCGTGCAGATG | | | | SL2.40ch06:31039807..31039825 | |
