# Supplementary material for: The Tomato Yellow Leaf Curl Virus Resistance Genes Ty-1 and Ty-3 Are Allelic and Code for DFDGD-Class RNA–Dependent RNA Polymerases
Source: PLoS Genet. 2013 Mar 28;9(3):e1003399. doi: 10.1371/journal.pgen.1003399 (PMC3610679; doi:10.1371/journal.pgen.1003399)
Supplement: Table S6 — Primers used in this study. (PDF) [file pgen.1003399.s012.pdf]

Supplemental table 6. Primers used in this study

Primers used to make the TRV2 VIGS constructs

		**Sequence 5'-3'**
TRV2-160	F	CACCATGGGAGCGATAACATTGAG
	R	CCCACTTCCACCACAACTCT
TRV2-180	F	CACCATGAAGACAAAAACTGCTTCTTTG
	R	ACCATTTTCATCCCGACAAA
TRV2-190	F	CACCGCTTGAGATGGGCTTATTGG
	R	TCTCCCAGGGCTCTCTGTAA
TRV2-PDS	F	CGGTCTAGAGGCACTCAACTTTATAAACC
	R	CGGGGATCCCTTCAGTTTTCTGTCAAACC

Primers used to amplify products that show Solyc06g051170, Solyc06g051180 and Solyc06g051190 together code for one gene (See supplemental figure 4)

	**Sequence 5'-3'**
F3	GCGTGGATCCTGCAAAGCGCA
R10	GGGAACAGCTGACCCCACTGGT
F7	CAGTAGCAGCTGACCTCGGGC
R7	TCGTGGACCTTGATGACGTGAATG
F6	CGGCACTGCATCAACAAAGGCG
R4	ACAGAGAGCCCTGGGAGATGACA
F4	CCATCATTGCAGGTTCCAATGGACT
R5	AGCAGGGGCAAAAACCACCG

Primers used for GeneRacer

	**Sequence 5'-3'**
F5	CTGAGGGCTTGCACAGGCCAAT
GeneRacer 5’	CGACTGGAGCACGAGGACACTGA
R3	GCAGCTGATAGCTGGCTGGC
GeneRacer 3’	GCTGTCAACGATACGCTACGTAACG

Primers used to amplify and TOPO clone the full length *Ty-1* and *Ty-3* . Start and stop codon are in bold

	**Sequence 5'-3'**
Ty-F7-CACC	CACCTTCAAGTATATACAGGAAAA**ATG**GGTGATCCG
Ty-R5	**CTA**GAGTATTTCCTGCAAAACCGATG
